# Controlling Internal Pore Sizes in Bicontinuous Polymeric Nanospheres**

**DOI:** 10.1002/anie.201408811

**Published:** 2015-01-16

**Authors:** Beulah E McKenzie, Heiner Friedrich, Maarten J M Wirix, Joël F de Visser, Olivia R Monaghan, Paul H H Bomans, Fabio Nudelman, Simon J Holder, Nico A J M Sommerdijk

**Affiliations:** Laboratory of Materials and Interface Chemistry and Soft Matter Cryo-TEM Research Unit, Eindhoven University of TechnologyP.O. Box 513, 5600 MB Eindhoven (The Netherlands); Functional Materials Group, School of Physical Sciences, University of KentCanterbury, Kent CT2 7NH (UK); Institute for Complex Molecular Systems, Eindhoven University of TechnologyP.O. Box 513, 5600 MB Eindhoven (The Netherlands)

**Keywords:** bicontinuous nanospheres, block copolymers, cryo-electron tomography, morphology, self-assembly

## Abstract

Complex polymeric nanospheres were formed in water from comb-like amphiphilic block copolymers. Their internal morphology was determined by three-dimensional cryo-electron tomographic analysis. Varying the polymer molecular weight (MW) and the hydrophilic block weight content allowed for fine control over the internal structure. Construction of a partial phase diagram allowed us to determine the criteria for the formation of bicontinuous polymer nanosphere (BPN), namely for copolymers with MW of up to 17 kDa and hydrophilic weight fractions of ≤0.25; and varying the organic solvent to water ratio used in their preparation allowed for control over nanosphere diameters from 70 to 460 nm. Significantly, altering the block copolymer hydrophilic–hydrophobic balance enabled control of the internal pore diameter of the BPNs from 10 to 19 nm.

In aqueous solution, block copolymer amphiphiles can organize into a variety of morphologies such as spherical and cylindrical micelles, vesicles,[1] helices,[2] and toroids,[3] but the formation of assemblies with more complex internal and surface structures has also been achieved.[4] Of particular interest are polymeric nanoparticles with bicontinuous internal structure,[5] within which a twisted network of the hydrophobic phase intertwines with that of the hydrated hydrophilic moiety. These bicontinuous polymer nanospheres (BPNs), similar to lipid cubosomes,[6] hold promise for applications in controlled release systems.[7] For example, we have shown in earlier work that encapsulation and temperature-controlled release of hydrophobic fluorescent molecules solvated in the PODMA domains can be achieved with poly(ethylene oxide)-*b*-poly(octadecyl methacrylate) (PEO-*b*-PODMA) BPNs.[7a] BPNs also show promise for use as templates for the formation of mesoporous materials, because the pores are accessible from the external aqueous medium[7c] and can potentially be infiltrated by aqueous mineral precursor to template the mineral growth.[8] Moreover, in contrast to their inorganic counterparts, e.g., based on silica,[9] they allow tailoring of the chemical structure,[10] and can show stimuli-responsive behavior.[7] However, despite their promise, no design criteria are yet available for the construction of BPNs with well-defined pores and tunable, predetermined pore sizes. BPNs have been reported in experimental studies for block copolymer amphiphiles with widely varying chemical structures and macromolecular architectures, such as linear,[5j] comb,[5a, 7c] and triblock copolymers[5b–d, i] as well as dendrimers;[5e] and from different preparation protocols. Additionally, simulations predict that it should be possible to tune the internal morphology of these nanospheres by changing the hydrophilic–hydrophobic balance of the block copolymer through variation of the PEO weight fraction (*f*) of the polymer.[11]

Here, we use a series of PEO-*b*-PODMA diblock copolymers with different molecular weight (MW) and varied *f* to explore the morphological variation inside polymer nanospheres. The internal morphologies were analyzed and resolved by 3D imaging using cryo-electron tomography (cryoET, 3D cryoTEM), and the resulting partial phase diagram showed that BPNs form at relatively low MW (≤17 kDa) and PEO content (*f*≤0.25). In addition, besides controlling the outer diameter of the nanospheres through varying the THF/water ratio in the preparation procedure (Figure 1), we can also tailor the diameter of the internal pores of the BPNs by varying the relative PEO content of the polymer in synthesis. Hence, we present here the first example of BPNs with tunable size and internal pore dimensions.

**Figure 1:**
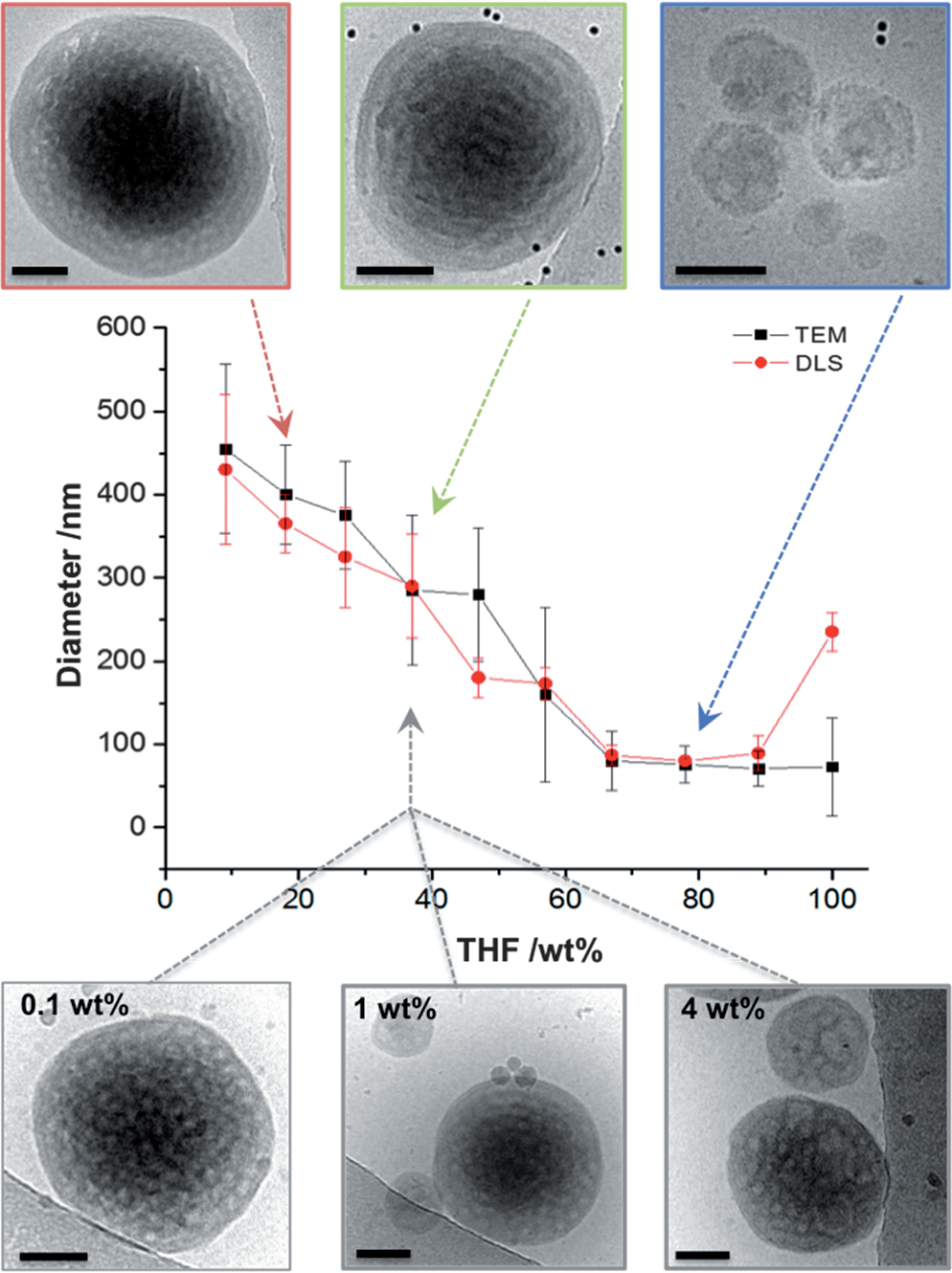
Graph showing the variation in particle diameter (as measured by TEM and DLS) with changing THF wt % content of the starting solution for dispersions formed from a single block copolymer that forms BPNs (PEO_47_-*b*-PODMA_20_; *f*=0.25). CryoTEM images of BPNs from different dispersions are shown to illustrate the difference in sizes. TEM sizes quoted are from an average of 50 particles. The lower row shows BPNs formed at 37 wt % THF at different concentrations of PEO_47_-*b*-PODMA_20_ in solution. Scale bars represent 100 nm.

A series of PEO-*b*-PODMA block copolymers was synthesized from PEO macroinitiators (MW ca. 2.0 and 5.0 kDa) by atom transfer radical polymerization (ATRP; Supporting Information (SI), Table 1).[12] The resulting block copolymers had low polydispersities (PDI=1.11–1.26) and varied in MW (6.7–25.8 kDa) as well as in PEO relative weight content (*f*=0.07–0.47). Dispersions of polymeric assemblies (1 g L^−1^) were obtained by dissolution of the block copolymer in tetrahydrofuran (THF)—a nonselective solvent—followed by the slow addition of water (nanoprecipitation), and subsequent dialysis for 24 h.

Dispersions of all copolymers were vitrified and analyzed by 2D cryoTEM, with in total 40 cryo-electron tomograms (3D cryoTEM) and computer-aided visualizations (segmentations), from which a partial phase diagram was constructed (Figure 2; SI, Figures S1–S16). This analysis revealed that a) inverse micellar structures form at high MW and low PEO content (MW>25 kDa; *f*=0.07); b) vesicular structures form at high MW and intermediate PEO contents (MW>19 kDa; 0.25≤*f*≤0.31); c, d) at lower MW and intermediate PEO content (*f*=0.22–0.34) multi-lamellar morphologies coexist with BPNs; e) BPNS form from polymers with relatively low MW and low PEO content (MW≤17 kDa, *f*≤0.25), and f) at the highest PEO contents (*f*=0.47) spherical micelles are formed. The 2D cryoTEM images for nanospheres of all of the phase diagram block copolymers can be found in the SI (Figures S1–S13). It is worth noting that cylindrical micelles (mixed with spherical micelles) could only be achieved for block copolymers with *f*=0.47 by increasing the copolymer concentration from 1 g L^−1^ to 5 g L^−1^ (Figure S5).

**Figure 2:**
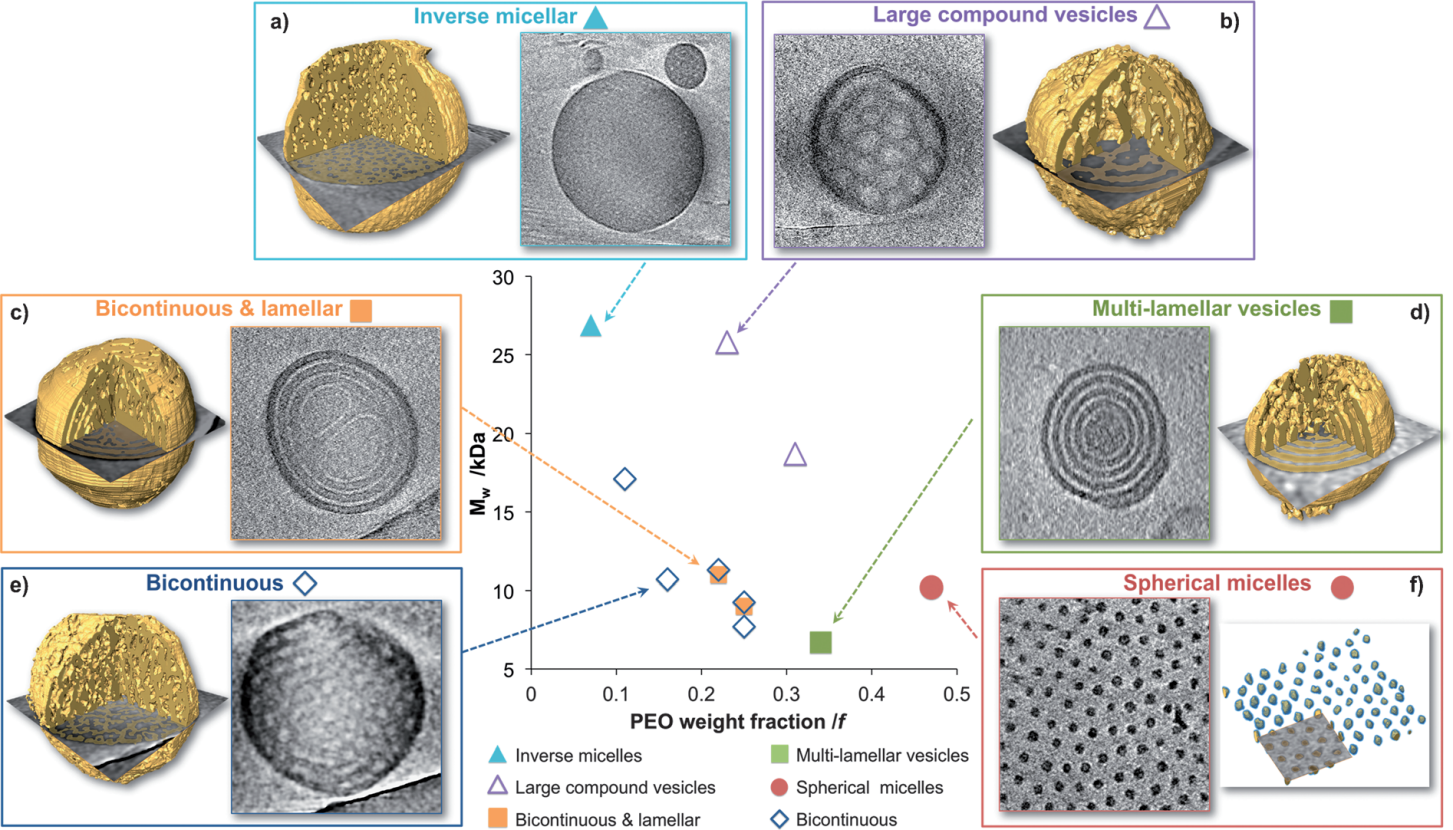
Partial phase diagram of the self-assembly behavior of PEO-*b*-PODMA block copolymers with corresponding slices through the 3D reconstructions, and computer-aided visualizations from the reconstructed tomograms (segmentations in yellow) that show the different morphologies. The computer-aided visualizations were conducted by combining the use of a mathematical filter to reduce noise and an adaptive threshold to segment the hydrophobic PODMA phase (shown in yellow). The compositions, MW parameters, and corresponding morphologies of all of the block copolymers are outlined in the SI, Tables S1 and S2.

The ability to tailor not only the internal morphology but also the sizes of the nanospheres and their internal features is of significant importance when considering their possible applications. For a single PEO-*b*-PODMA block copolymer forming BPNs, the average size of the resulting nanoparticles could be tuned between 70±30 nm and 460±100 nm by modulating the PODMA–solvent interfacial energy through variation of the volume ratio of the initial THF/water solution (Figure 1), whilst keeping the total amount of polymer constant (1 g L^−1^ in all cases) and preserving the internal morphology (SI, Table 4 and Figures S7 and S8). Additionally, stable BPN dispersions could be obtained from a single block copolymer (PEO_47_-*b*-PODMA_20_; *f*=0.25) upon increasing the polymer concentration from 0.1–4 wt %, whilst preserving the bicontinuous morphology (Figure 1).

CryoET is the most powerful tool for studying the internal structure of polymer nanoparticles as it enables complete 3D analysis of the nanosphere volume.[13] 3D visualization is particularly important for characterizing nanospheres with small and superimposing features (in the present case, nanospheres with inverse micellar morphology). In utilizing this for the analysis of BPNs, we observe that for the nanoparticles formed from polymers with 0.07≤*f*≤0.25 the internal pore connectivity decreases with decreasing PEO content. At *f*=0.07 an inverse micellar phase exists in which spherical PEO/water compartments are encapsulated within a PODMA matrix. When *f*=0.11 the nanospheres exhibit bicontinuity, with the inverse micellar phase persisting in some regions. At 0.16≤*f*≤0.25, the nanospheres consistently show bicontinuity, although on occasion the centers of the spheres contain denser regions with limited interconnectivity (Figure 3).

**Figure 3:**
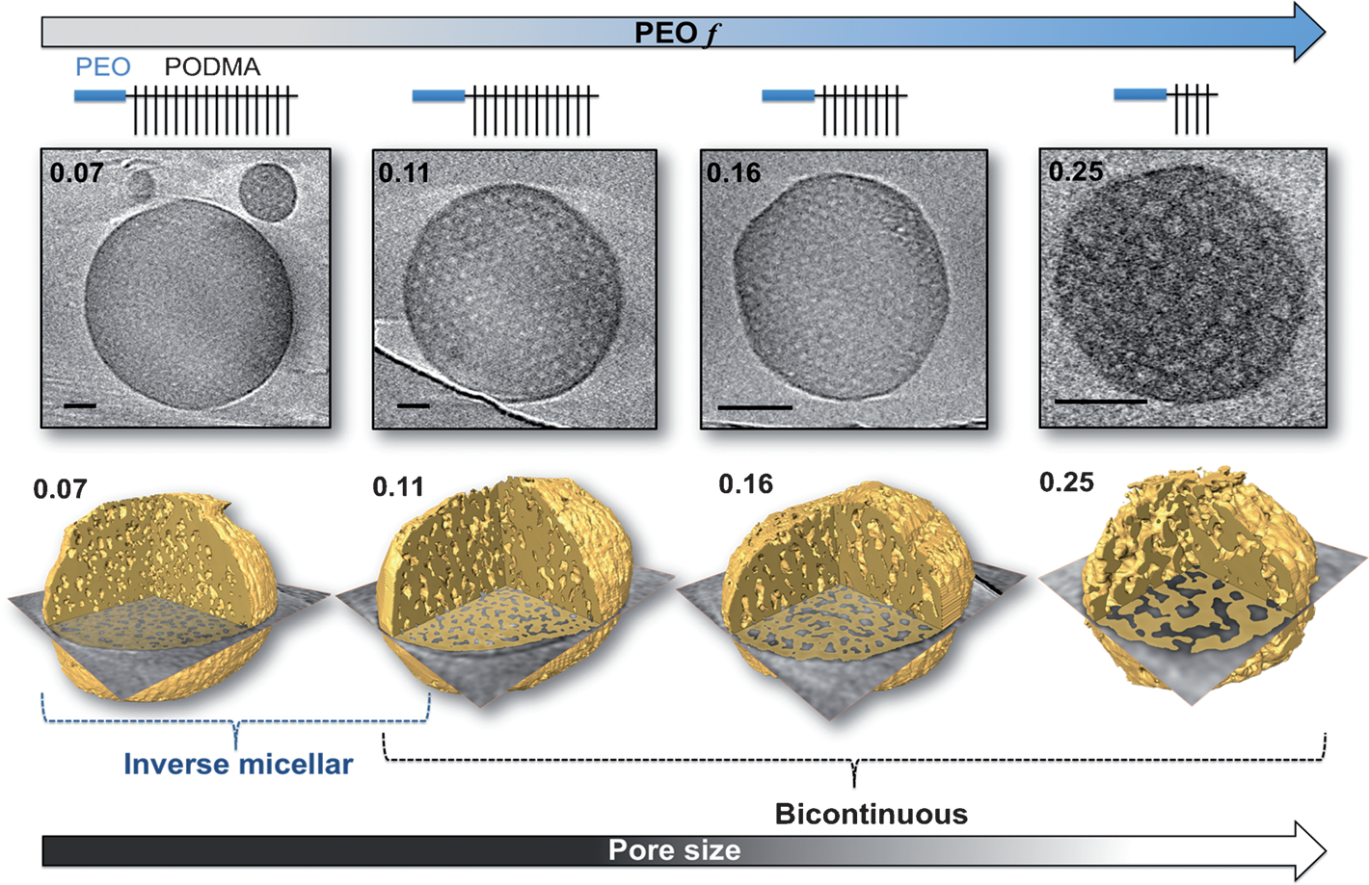
Slices through the 3D reconstruction of BPNs from different PEO-*b*-PODMA block copolymers, with corresponding cartoon representations of the polymer structure. The dark regions correspond to PODMA and the light regions correspond to the hydrated PEO pores. Scale bars represent 100 nm. The lower row shows segmentations of the reconstructed tomograms with the slice overlaid, revealing the internal morphology. The yellow parts correspond to the PODMA phase.

Previously the bicontinuous phase has been reported to be a metastable morphology upon transition between thermodynamically preferred morphologies.[5f,i,^14^] However, as we have demonstrated previously, cycling the temperature above and below the melting point of PODMA (22 °C) clearly shows a reversible phase transition attributable to the melting transition of the PODMA blocks.[7c] In all cases to date the bicontinuous phase reforms upon cooling below the crystallization point. In the present case, we observe no change in the PEO-*b*-PODMA BPN morphology even after 20 months (Figure S8), which underlines the colloidal stability of the dispersions. In the present case, the BPNs are formed from highly asymmetric PEO-*b*-PODMA comb-like block copolymers with low hydrophilic content (*f*≤0.25), which promotes the formation of morphologies with inverse curvature; however, we have recently presented BPNs formed from simple linear diblock copolymers of PEO-poly(*n*-butyl methacrylate) (PEO-*b*-PBMA) suggesting that the comb-like structural feature of the block copolymer is not a prerequisite for bicontinuous phase formation.[5j] The formation of a multilamellar (“onion-like”) phase was observed for the PEO-*b*-PBMA block copolymers as well as the bicontinuous phase, dependent upon preparation conditions (notably the solvent: THF versus dioxane). Therefore, whilst we must allow for the possibility that the bicontinuous phase may not be the most thermodynamically stable phase for the PEO-*b*-PODMA block copolymer, it is kinetically and thermodynamically stable under the conditions observed (in water from 4 to 25 °C and for time periods of up to 20 months).

The partial phase diagram shows how the variation of the block copolymer composition changes the internal morphology of the nanospheres from inverse morphologies to lamellar to spherical micelles (as predicted by packing parameter model).[15] BPNs form at MW≤17 kDa, *f*≤0.25. If the BPNs were metastable, then we would expect changes in the morphology upon changing the polymer concentration in solution or the amount of cosolvent in the preparation (more cosolvent would aid the formation of the “real” thermodynamically preferred phase). It is notable that we do not observe morphological changes with changes in either of these preparation condition variables (Figure 1). It is also worth noting that many BPN dispersions are formed in the presence of additives[5c,d,h] and a nonselective cosolvent such as THF,[5c,g] dimethylformamide (DMF),[5b,f] dimethyl sulfoxide (DMSO),[5a] and ethanol.[5e] The BPNs presented here form in THF/water mixtures, in which THF is a nonselective solvent that is able to solubilize both blocks of the copolymer. This is necessary to confer the bulky high molecular weight polymers with the conformational mobility needed to form ordered assemblies, and to minimize the rapid formation of kinetically trapped non-equilibrium structures due to the increasingly poor quality of the solvent when water is added. The solubility parameters of PEO and PODMA (10.5 and 7.8 (cal cm^−3^)^1/2^, respectively) are much closer to that of THF (9.1 (cal cm^−3^)^1/2^) compared with water (23 (cal cm^−3^)^1/2^),[16] suggesting that both PEO and PODMA are preferentially solubilized in THF as opposed to water and may support previous proposals that, similar to other porous morphologies, BPNs originate from polymer-rich THF droplets that form in water.[5b,c, 17] A detailed study of the formation process will be addressed in a forthcoming full paper.

Analysis of the internal domains showed that the internal pore size of the BPNs increased from 10±2 nm to 19±3 nm with an increase in *f* from 0.11 to 0.25 (i.e., decreasing DP_PODMA_) (Figures 3, 4, and S16). Moreover, the increased pore size of the BPNs was determined only by the relative PEO content and not by the absolute MW of the PEO. For example, BPNs from PEO_39_-*b*-PODMA_17_ and PEO_47_-*b*-PODMA_20_ gave pore diameters of 19±2 nm and 17±3 nm, respectively, at a constant relative PEO content (*f*=0.25), despite the MW of the PEO being larger by 30 %. Reconstructions of the nanospheres showed abundant openings in the BPNs’ outer membrane, which leave the inner pore volume accessible from the external medium. Controlling the pore size by simply changing the relative hydrophilic content presents an exciting possibility when considering the application of the BPNs as templates for the infiltration and organization of inorganic materials to form hybrid materials. It is important to note that in general for micellar and vesicular structures, the dimensions of the hydrophobic domains scale positively with the DP of the hydrophobic block.[18] In contrast with this, we observe that for the BPNs the PODMA pore wall thickness remains almost unchanged and varies only between 9.5±2 nm and 11.5±3 nm, even when the DP was increased by three times (Figure S18). These observations suggest that the polymer chains pack in a complex fashion to maintain the bicontinuous morphology. Computational investigation of the structures will be undertaken in forthcoming studies in the hope of providing a more detailed molecular understanding of the complex self-assembly behavior.

**Figure 4:**
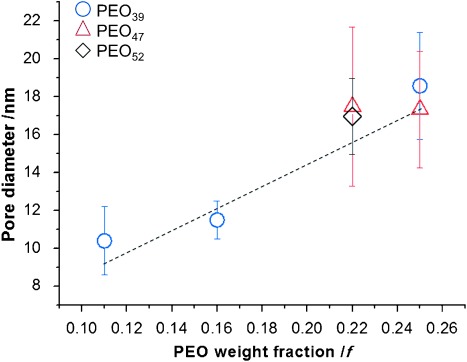
Graph showing the diameter of the pores of BPNs formed from block copolymers from different PEO macroinitiators as a function of the PEO weight fraction (*f*) of the block copolymer.

In summary, through 3D analysis using cryoET we have been able to resolve the internal morphology of complex polymeric nanospheres and demonstrate that control of the internal structure can be readily achieved by changing the overall MW and relative hydrophilic content of the composite polymer. Hereby, we show that—in line with earlier computational predictions[11]—PEO-*b*-PODMA BPNs form in a distinct region of the compositional phase diagram. Stable dispersions with concentrations of 0.1–4.0 wt % can be prepared for which both the outer diameter as well as the diameter of the internal pores can be tailored by changing the relative volume of the starting solution and by tuning the hydrophobic–hydrophilic balance of the polymer blocks, respectively. Considering that BPNs can be obtained from a variety of block copolymer architectures, the present demonstration that they can be tailored and designed opens the way for the use of these BPNs in a variety of applications such as controlled release vectors or as templates for the synthesis of inorganic and hybrid materials.

## Experimental Section

Block copolymer synthesis: PODMA was copolymerized with a presynthesized PEO macroinitiator through atom transfer radical polymerization (ATRP) according to literature procedures.[12a] Typically, a bromo-functionalized PEO macroinitiator, *N*-(*n*-octyl)-2-pyridyl(methanime) ligand, ODMA, and copper(I) bromide were dissolved in an isopropanol/xylene solvent mixture in a Schlenk tube and degassed with dry N_2_ for 30 min before placing in an oil bath at 95 °C for 12 h. The polymer was dissolved in THF and passed through an alumina column and precipitated from methanol.

Preparation of polymer dispersions: Typically, 10 mg of polymer was dissolved in a predetermined volume of THF at 35 °C, and water added at a rate of 0.067 mL min^−1^ until a final polymer concentration of 1 g l^−1^ was obtained. The resulting dispersion was dialyzed against water at 35 °C for 24 h and stored at 4 °C before analysis.

Visualization of polymer nanospheres: Samples were imaged in 2D and 3D (cryo electron tomography) in a FEI CryoTitan operated at 300 kV and equipped with a field emission gun (FEG). Samples were prepared on 200 mesh Cu TEM grids. In general, 3 μL of the dispersion mixed with a colloidal gold solution (at 4 °C) was applied to the hydrophilized TEM grid, blotted and vitrified in an automated vitrification robot (FEI Vitrobot™ Mark III) by plunging into liquid ethane. Tomographic tilt series acquisition was performed with Inspect3D software (FEI Company). Alignment and reconstruction of the series were carried out using IMOD[19] and denoised prior to visualization. Measurements for the BPNs’ internal domains were obtained by analyzing slices through the 3D reconstructions using inhouse MATLAB scripts. Segmentations were completed in Avizo® Fire, a 3D analysis software. The figures show a volume rendering of the nanoparticles combined with an isosurface (the surface between the PEO and PODMA phases), and an overlay of the orthoslice taken from the respective denoised reconstruction.
