# Supplementary material for: Controlling Internal Pore Sizes in Bicontinuous Polymeric Nanospheres
Source: Angew Chem Int Ed Engl. 2015 Jan 16;54(8):2457–61. doi: 10.1002/anie.201408811 (PMC4471611; doi:10.1002/anie.201408811)
Supplement: Supplementary file 1 [file anie0054-2457-sd1.pdf]

Angewandte Chemie
Eine Zeitschrift der Gesellschaft Deutscher Chemiker

Supporting Information

© Wiley-VCH 2015

69451 Weinheim, Germany

Controlling Internal Pore Sizes in Bicontinuous Polymeric Nanospheres**

Beulah E. McKenzie, Heiner Friedrich, Maarten J. M. Wirix, Joël F. de Visser, Olivia R. Monaghan, Paul H. H. Bomans, Fabio Nudelman, Simon J. Holder, and Nico A. J. M. Sommerdijk**

anie_201408811_sm_miscellaneous_information.pdf

Supporting Information

Table of Contents

1. Experimental Section…………………………………………………..……………………...3
2. Syntheses…………………………………………..………………………………………..6
3. Block copolymer molecular weight parameters……………………………….………...8
4. Procedures for the preparations of the aqueous dispersions……...…………………………9
5. CryoTEM and DLS measurements……………………...………………………………12
6. 3D cryo electron tomography……………………………………………...……………20
7. Size measurements……………………..……………………...……………………..….23

Experimental Section

Materials and Apparatus

Poly (ethylene glycol) methyl ether (molecular weights ca. 2.0 and 5.0 kDa), 2-bromoisobutyryl bromide (98%), dimethylamino pyridine (99%), triethylamine (99%), octadecyl methacrylate (ODMA) and copper (I) bromide (98%) were all purchased without further purification from Sigma-Aldrich. Aluminium oxide (activated, neutral, for column chromatography 50-200μm) and magnesium sulphate (97%, anhydrous) were purchased from Acros Organics. Sodium bicarbonate (analytical reagent grade) was purchased from Fisher Scientific. Hydrochloric acid (35.4%; sp. gr. 1.18) was purchased from BDH Chemicals. N-(*n*-octyl)-2-pyridyl(methanime) was synthesized according to literature procedures.[1] Methanol (analytical reagent grade), tetrahydrofuran (analytical reagent grade), isopropanol, ethanol (analytical reagent) and water (HPLC gradient grade) were all purchased from Fisher Scientific. All polymerization reactions were conducted under an inert atmosphere of dry nitrogen using standard Schlenk line techniques.

Dichloromethane (analytical reagent grade) was purchased from Fisher Scientific, and before use was dried and distilled over calcium hydride. σ-xylene was purchased from BDH Lab Supplies. The deuterated solvent D1-chloroform (99.8%) was used as received from Cambridge Isotope Laboratories Incorporated.

Characterization

^{1}H NMR spectra were recorded using a JEOL GX-270 FT spectrometer (270 MHz) at 25°C in solutions of deuterated chloroform ($CDCl_3$). Molecular weight averages were calculated via size exclusion chromatography (SEC) using two 5μm mixed C PLgel columns at 40°C, calibrated using poly(methyl methacrylate) standards. The samples in THF were detected by a Shodex RI-101 refractive index detector. Infra-red spectra were collected using a Thermo Nicolet Avatar 360 FT-IR spectrometer.

Dialysis

Dialyses were conducted in miliQ water using a 5ml QuixSep Dialyzer™ (Orange Scientific) covered with a dialysis membrane (Medicell International Ltd., 36/32" diameter) with a cut off at approximately 12.0-14.0 kDa. Water (HPLC Grade, Fisher Scientific) and THF (Analytical grade, Fisher Scientific) were filtered using Sartorius Minisart 0.45 μm filters before use.

In a typical procedure, 10 mg of block copolymer was dissolved in 4 ml THF and left stirring in an oil bath at 35 °C. 6 ml water (HPLC Grade, filtered) was added drop-wise to the stirring solution over 90 mins. The solution was transferred to the dialyzer, sealed with the dialysis membrane and immersed in 4 L of stirring 35 °C miliQ water for 24 h to displace the THF. During this time, the water was replaced twice.

Optical and Size Measurements

Dry transmission electron microscopy (TEM) was carried out using a JEOL JEM (200-FX) machine, operating at 120 kV. 20 μl of the dialyzed sample was deposited onto a carbon-coated copper grid (200 mesh) left for 30 s and removed via suction. The grid was then stained with a solution of 5 % uranyl acetate and 1 % acetic acid. 20 μl of this solution was

deposited onto the grid and removed after 5 s. Excess solution was dabbed away using filter paper. Particle size measurements were carried out using an electron density profile tool, and averages were determined from 50 particles. Dynamic light scattering measurements were carried out on a Malvern High Performance Particle Sizer (Nano Zetasizer HPPS HPP5001) with a laser at a wavelength of 633 nm. 1 ml of the dialyzed solution was taken, filtered using a 1.2 μm filter and placed in a clean cuvette. The desired temperature was set (4 °C) and the sample left at this temperature for 15 mins before the runs were conducted. The particle sizes, size distributions and polydispersity for each aggregate solution were analyzed. At each temperature, ten size readings were obtained and the quoted size values are an average of these results.

Cryo TEM and Cryo ET sample preparation

CryoTEM samples for 2D and 3D imaging were prepared on 200 mesh Cu TEM grids containing a hydrophilized R2/2 Quantifoil carbon support film (Quantifoil Micro Tools GmbH, Jena, Germany). The carbon support film was hydrophilized by surface plasma treatment (glow discharge) in a Cressington 208 carbon coater directly prior to use. In general, 27 μl of the dialyzed solution equilibrated to the desired temperature (4 °C), was mixed with 3 μl of a colloidal gold solution (4 °C, 5 nm diameter, PAG coated). Subsequently, 3 μl of this mixture was applied to the hydrophilized TEM grid, blotted and vitrified in an automated vitrification robot (FEI Vitrobot^{TM} Mark III) by plunging into liquid ethane. This process was performed with the environmental chamber of the Vitrobot conditioned to 100 % humidity and 4 °C to prevent temperature and drying artefacts.

CryoTEM and Cryo ET imaging

Samples were imaged in two dimensions and three dimensions (cryo electron tomography) in a FEI CryoTitan operated at 300 kV and equipped with a field emission gun (FEG). For imaging low-dose mode and underfocus conditions were used (-5 μm to -50 μm depending on the magnification) with a 70 μm objective aperture inserted in the beam path. Images were recorded zero-loss filtered with a post column Gatan Energy Filter (20 eV energy window) using a 2k x 2k Gatan slow scan CCD camera. Tomographic tilt series acquisition was performed with Inspect 3D software (FEI Company). Alignment and reconstruction was carried out in IMOD[2] using SIRT with 10 iterations. Subsequently data were denoised by nonlinear anisotropic diffusion prior to visualization.[3]

Typical electron tomography acquisition conditions:

Temperature at vitrification: 4 °C
Typical angular sampling: 0° to +64 and to -64° at 1° or 2° increments
Magnification: 19000 × or 24000 ×
Defocus: -5 to -10 μm
Total electron dose: 60 to 100 $e^{-}.Å^{-2}$
Pixel size at 19000 x : 0.463 nm for images; 0.93 nm voxel size in the corresponding tomogram
Pixel size at 24000 x : 0.370 nm for images; 0.74 nm voxel size in the corresponding tomogram

Segmentation

Segmentation of the reconstructed tomograms was conducted with several steps to reduce noise and specifically segment out the features that were observed and interpreted visually from the slices of the reconstructed tomograms. Noise reduction was implemented using a nonlinear anisotropic diffusion (NAD) filter in IMOD.[2-3] The rest of the segmentation was carried out in Avizo® Fire, a 3D analysis software. A mask was generated by manually tracking the edges of the sphere and subsequent interpolation over a few slices. This mask was then applied to the tomogram to select only the sphere; the background surrounding the sphere was made transparent. Subsequently, the dark regions in the sphere were segmented out (PODMA) by using a tophat filter and threshold to segment out the local structures, whilst ignoring the large-scale intensity variations. The threshold was chosen by visually judging the best match of the segmentation with the slices of the tomogram. Optionally, in some volumes a morphological opening, closing or erosion was done to highlight certain features that were observed in the slices of the tomogram. The figures show a volume rendering combined with an isosurface and an orthoslice. The volume rendering in yellow is of the PODMA phase, whilst the water/PEO phase is transparent. The isosurface was generated on the border of the PODMA phase and the water/PEO phase. The orthoslice was taken from the NAD filtered reconstruction and overlaid with the local segmentation in yellow. A full summary of the segmentations including the morphological operations and the ball sizes that were used as structuring elements in pixels are given in the table below.

**Morphology**	**Tophat**	**Opening**	**Closing**	**Erosion**	**Dilation**
Bicontinuous (0.25)	12	2	2		
Bicontinuous and lamellar (0.22)	5	2	3		
Inverse micellar (0.07)	5		3	1	
Large compound vesicles (0.23)	12	5	6		
Lamellar (0.34)	7				
Spherical micelles (0.47)	5	2	3		3 (blue)
Bicontinuous (0.11)	5				
Bicontinuous (0.16)	5				
Bicontinuous (0.25)	15				

Syntheses

Preparation of Ethylene Glycol Methyl 2-Bromo 2-Methyl Propanoate (PEO Macrointiator)

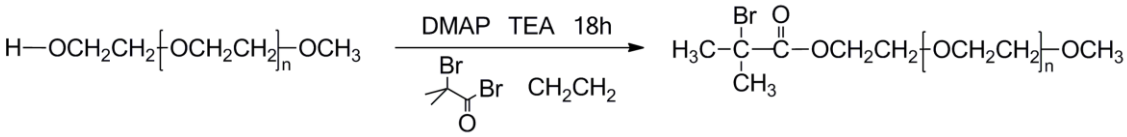

A modification of a literature method was used[4] and was typically carried out as follows: a solution of PEGME (M_n ca. 2.0 kDa) (10 g, 5 mmol) in 50ml dichloromethane was added dropwise to a stirred mixture of 2-bromoisobutyryl bromide (1.24 ml, 10 mmol), triethylamine (1.4 ml, 10 mmol), and dimethylamino pyridine (1.22 g, 10 mmol) in dichloromethane at 0 °C for 1 h under nitrogen. The solution was stirred for a further 18 h at room temperature. A third of the reaction solvent was evaporated off and the resultant yellow precipitate was filtered off. The remaining solution was made up to 100 ml with dichloromethane and transferred to a separating funnel. The solution was washed several times with a saturated sodium bicarbonate solution and then with a 10 % hydrochloric acid solution. The organic layer was collected and dried over anhydrous magnesium sulphate for an hour, filtered and the dichloromethane evaporated off. Finally, the resultant yellow solid was left under vacuum at 50 °C for two hours. Yields ranged from 50-95 %. The structure was confirmed using ^{1}H, ^{13}C NMR and Fourier Transform Infrared (FTIR) spectroscopy, and the molecular weight parameters determined using size exclusion chromatography (SEC).

^{1}H NMR (270 MHz, $CDCl_3$, ppm) δ: 1.80 (singlet, 6H, $(CH_3)_2C$-), 3.36 (singlet, 3H, $-OCH_3$), 3.59 (broad peak, 4H $-OCH_2CH_2$-), 3.84 (triplet, 2H, $–CH_2O$-), 4.28 (triplet, 2H, $O=COCH_2$-).
^{13}C NMR (270 MHz, $CDCl_3$, ppm) δ: 30.8 [$(CH_3)_2$], 55.7 (C-Br), 59.0 ($-OCH_3$), 65.1 ($O=COCH_2$-), 68.7 ($-CH_2O$-), 70.6 ($-CH_2CH_2O$-), 71.9 ($-CH_2-OCH_3$), 171.6 (–C=O).
FTIR (cm^{-1}): 2883 (strong peak C-H stretches), 1735 (moderate peak C=O), 1147 (weak peak C-O ester stretch), 1100 (strong peak C-O ether stretches), 528 (weak peak C-Br).

Synthesis of PEO-PODMA block copolymers via ATRP

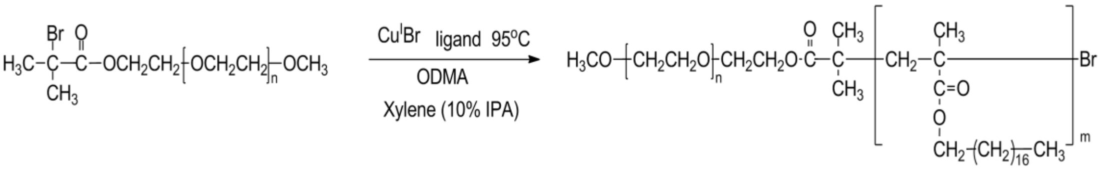

A modified literature synthesis was conducted as follows:[1a] The PEO macroinitiator (0.84 g, 0.44 mmol) was placed in a Schlenk tube along with a magnetic stirrer, and dissolved in 2 ml of a solution of xylene:IPA (9:1 vol/vol). To this solution were added octadecyl methacrylate (also dissolved in 2 ml xylene:IPA (9:1)) (2.5 g, 7.4 mmol), N-(*n*-octyl)-2-pyridyl(methanime) (0.19 g, 0.88 mmol) and Cu^IBr (0.06 g, 0.44 mmol). The Schlenk tube was then sealed and the solution degassed with nitrogen for 30 mins. The reaction mixture was stirred at 95 °C for 12 h under N_2. The reaction mixture was then exposed to air, diluted in THF and run through an alumina column. The polymer was isolated by precipitation from the THF solution into methanol and yields ranged from 40-90 %. The structure was

confirmed using ^{1}H, ^{13}C NMR and FTIR spectroscopy, and molecular weight parameters determined using SEC.

^{1}H NMR (270 MHz, $CDCl_3$, ppm) δ: 0.88 (broad peak, 3H, –$(CH_2)_{17}$-CH_3), 1.03-1.4 (broad peaks, CH_3-C-CH_2- and -CH_2-$(CH_2)_{15}$-), 1.26 (broad peak, 30H -$(CH_2)_{15}$-), 1.53-2.10 (broad peaks, CH_3-C-CH_2-), 3.38 (singlet, 3H –OCH_3 PEO), 3.64 (broad peak, 4H –(OCH_2CH_2)-PEO), 3.91 (broad peak, 2H, O=CO-CH_2).
^{13}C NMR ($CDCl_3$, ppm) δ: 14.1 (-**C**H_3); 18.3 (CH_3-C-CH_2-); 26.0 (-$OCH_2CH_2CH_2$); 28.1 (-OCH_2CH_2-); 29.4 (-$CH_2(CH_2)_{10}CH_2$-); 29.7 (-(**C**$H_2)_{10}$-); 31.9 (-$CH_2CH_2CH_3$); 64.9 (-OCH_2-); 70.5 (-(O**C**H_2**C**H_2)-).
FTIR (cm^{-1}): 2916-2849 (strong peaks C-H stretches), 1727 (strong peak, C=O), 1146 (moderate peak C-O stretches).

Table S1: Molecular weight parameters of PEO initiators and PEO-PODMA block copolymers determined by ^{1}H NMR and SEC. Colour shading corresponds to grouping of block copolymers synthesised from the same PEO macroinitiator.

Structure	$M_n{}^a$ /kDa	$M_n{}^b$ /kDa	$M_w{}^b$ /kDa	$M_w/M_n{}^b$ /kDa	PEO wt fraction / f^a
PEO_{39}	1.90	2.80	3.00	1.09	-
PEO_{47}	2.30	2.70	2.80	1.05	-
PEO_{49}	2.38	3.60	3.90	1.06	-
PEO_{52}	2.50	2.55	2.70	1.07	-
PEO_{112}	5.20	6.30	6.80	1.08	-
PEO_{127}	5.80	7.30	5.50	1.07	-
PEO_{39}-*b*-$PODMA_{74}$	27.00	33.20	43.20	1.30	0.07
PEO_{39}-*b*-$PODMA_{47}$	17.80	19.30	23.50	1.22	0.11
PEO_{39}-*b*-$PODMA_{26}$	10.70	12.80	15.40	1.20	0.16
PEO_{39}-*b*-$PODMA_{17}$	7.70	10.80	11.90	1.11	0.25
PEO_{47}-*b*-$PODMA_{26}$	11.10	10.40	12.70	1.22	0.22
PEO_{47}-*b*-$PODMA_{20}$	9.00	10.90	13.70	1.26	0.25
PEO_{47}-*b*-$PODMA_{13}$	6.70	7.20	8.90	1.23	0.34
PEO_{49}-*b*-$PODMA_{21}$	9.50	15.40	19.90	1.29	0.25
PEO_{52}-*b*-$PODMA_{26}$	11.30	10.40	12.90	1.25	0.22
PEO_{112}-*b*-$PODMA_{15}$	10.20	10.00	11.50	1.15	0.47
PEO_{127}-*b*-$PODMA_{59}$	25.80	16.60	20.10	1.21	0.23
PEO_{127}-*b*-$PODMA_{38}$	18.70	15.60	19.40	1.24	0.31

[a] Obtained from ^{1}H NMR in $CDCl_3$

[b] Obtained from SEC in THF relative to PMMA standards.

Procedures for Preparation of the Aqueous Dispersions

Table S2: Variation of starting volume of THF in the dialysis preparation for each polymer. Each preparation was conducted at 35 °C. A cross denotes precipitation occurred; a tick denotes aggregation with no visible floc/ solid precipitation.

**Structure**	**PEO *f***	**DP_{ODMA}**	**THF Volume (ml)**								
			**9**	**8**	**7**	**6**	**5**	**4**	**3**	**2**	**1**
PEO_{39}-*b*-$PODMA_{74}$	0.07	74	✓	X	X	X	X	X	X	X	X
PEO_{39}-*b*-$PODMA_{47}$	0.11	47	✓	X	X	X	X	X	X	X	X
PEO_{39}-*b*-$PODMA_{26}$	0.16	26	✓	✓	✓	✓	✓	✓	X	X	X
PEO_{39}-*b*-$PODMA_{17}$	0.25	17	✓	✓	✓	✓	✓	✓	X	X	X
PEO_{112}-*b*-$PODMA_{15}$	0.47	15	✓	✓	✓	✓	✓	✓	✓	✓	✓

The minimal THF:water ratio required to prevent solid precipitation in the dispersion preparations was dependent upon DP_{PODMA} and the PEO weight fraction. Block copolymers with $DP_{PODMA} > 26$ and $f < 0.16$ required greater volumes of THF than did those with content of $DP_{PODMA} < 26$ and $f > 0.16$. However, varying the initial THF:water ratio for the preparations from a particular block copolymer yielded no morphological changes. Hence, the observed morphological differences between the different block copolymers can be attributed unambiguously to the polymeric composition and not to the preparation conditions.

Table S3: The final THF:water volume ratios used in the nanoprecipitation stage (before dialysis) to prepare the aggregate dispersions.

Structure	PEO *f*	DP_{PODMA}	$THF:H_2O$ volume ratio in water addition process (mL)
PEO_{39}-*b*-$PODMA_{74}$	0.07	74	9:1
PEO_{39}-*b*-$PODMA_{47}$	0.11	47	9:1
PEO_{39}-*b*-$PODMA_{26}$	0.16	26	4:6
PEO_{39}-*b*-$PODMA_{17}$	0.25	17	4:6
PEO_{47}-*b*-$PODMA_{26}$	0.22	26	4:6
PEO_{47}-*b*-$PODMA_{20}$	0.25	20	4:6
PEO_{47}-*b*-$PODMA_{13}$	0.34	13	4:6
PEO_{49}-*b*-$PODMA_{21}$	0.25	21	4:6
PEO_{52}-*b*-$PODMA_{26}$	0.22	26	4:6
PEO_{112}-*b*-$PODMA_{15}$	0.47	15	4:6
PEO_{127}-*b*-$PODMA_{59}$	0.23	59	9:1
PEO_{127}-*b*-$PODMA_{38}$	0.31	31	9:1

Table S4: The relative amounts of THF and water used in each water addition (nanoprecipitation) step when preparing bicontinuous nanosphere dispersions of PEO_{47}-*b*-$PODMA_{20}$ to alter particle diameter.

THF:water ratio (mL)	THF wt%
1:9	9
2:8	18
3:7	27
4:6	37
5:5	47
6:4	57
7:3	67
8:2	78
9:1	89
10:0	100

Table S5: Particle size characterization of nanospheres formed from different PEO-*b*-PODMA block copolymers. TEM sizes quoted are averages from measurement of 50 particles. Colour shading corresponds to grouping of block copolymers synthesised from the same PEO macroinitiator.

**Structure**	**M_n[a] / kDa**	**PEO *f***	**Morphology**	**Diameter / nm TEM**	**Diameter / nm DLS**	**PDI DLS**
PEO_{39}-*b*-$PODMA_{74}$	27.00	0.07	Inverse micellar	240 ±100	260 ±10	0.25
PEO_{39}-*b*-$PODMA_{47}$	17.80	0.11	Bicontinuous/ inverse micellar	220 ±150	140 ±2	0.18
PEO_{39}-*b*-$PODMA_{26}$	10.70	0.16	Bicontinuous	280 ±70	230 ±10	0.10
PEO_{39}-*b*-$PODMA_{17}$	7.70	0.25	Bicontinuous	200 ±80	280 ±30	0.31
PEO_{47}-*b*-$PODMA_{26}$	11.10	0.22	Bicontinuous/ lamellar	320 ±80	320 ±20	0.14
PEO_{47}-*b*-$PODMA_{20}$	9.00	0.25	Bicontinuous	285 ±90	300 ±60	0.33
PEO_{47}-*b*-$PODMA_{13}$	6.70	0.34	Bicontinuous and vesicular structures	150 ±50	150 ±10	0.23
PEO_{49}-*b*-$PODMA_{21}$	9.50	0.25	Bicontinuous	370 ±50	380 ±10	0.28
PEO_{52}-*b*-$PODMA_{26}$	11.30	0.22	Bicontinuous	300 ±50	310 ±80	0.38
PEO_{112}-*b*-$PODMA_{15}$	10.20	0.47	Spherical micelles	30 ±10	30 ±3	0.25
PEO_{127}-*b*-$PODMA_{59}$	25.80	0.23	Compound vesicles and vesicular structures	190 ±60	120 ±30	0.18
PEO_{127}-*b*-$PODMA_{38}$	18.70	0.31	Compound vesicles and vesicular structures	170 ±60	130 ±20	0.17

CryoTEM and DLS Measurements

All results are acquired from 0.1 wt% polymer dispersions unless otherwise stated. CryoTEM and DLS were conducted at 4 °C. In DLS figure legends, I_{ave}, N_{ave} and V_{ave} are the intensity, number and volume size average curves respectively. Black particles in cryoTEM micrographs are gold nanoparticles used as fiducial markers for electron tomography. All scale bars represent 100 nm.

Figure S1: CryoTEM images and the corresponding DLS size average curves for aggregates of PEO_{39}-*b*-$PODMA_{74}$ (f = 0.07), which forms inverse micellar nanospheres.

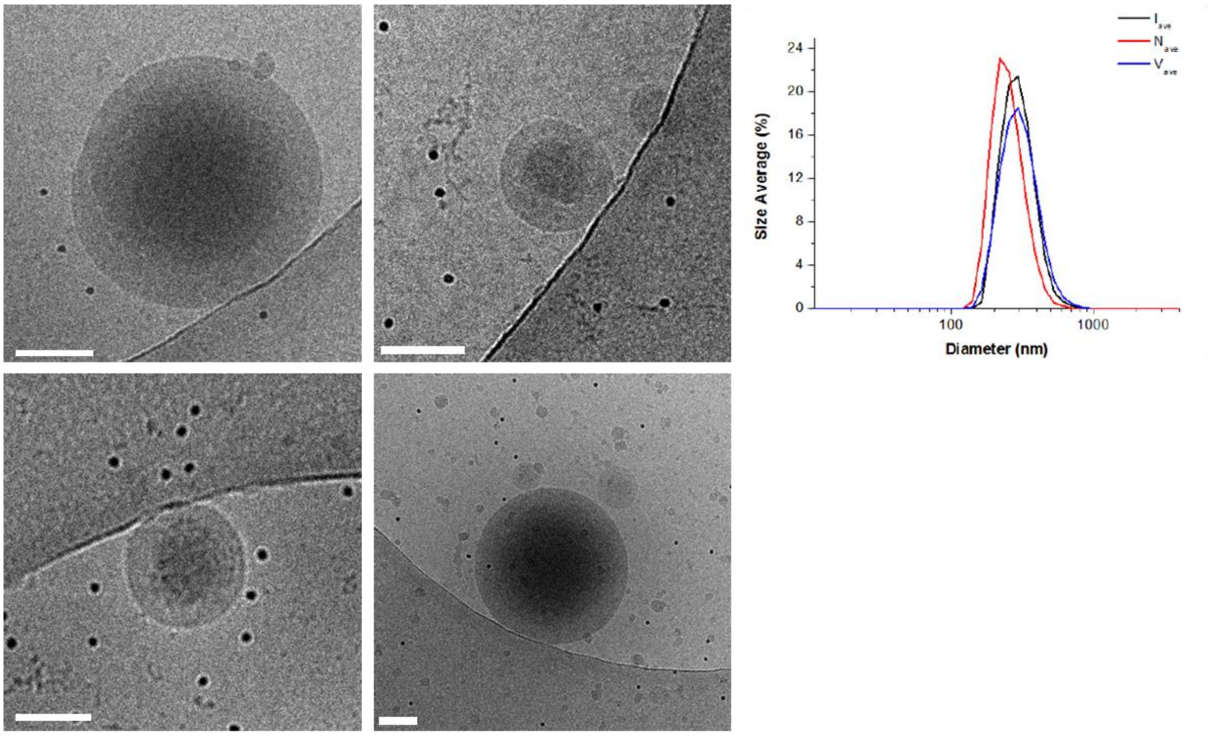

Figure S2: CryoTEM images and the corresponding DLS size average curves for aggregates of PEO_{39}-*b*-$PODMA_{47}$ (f = 0.11), which forms bicontinuous nanospheres.

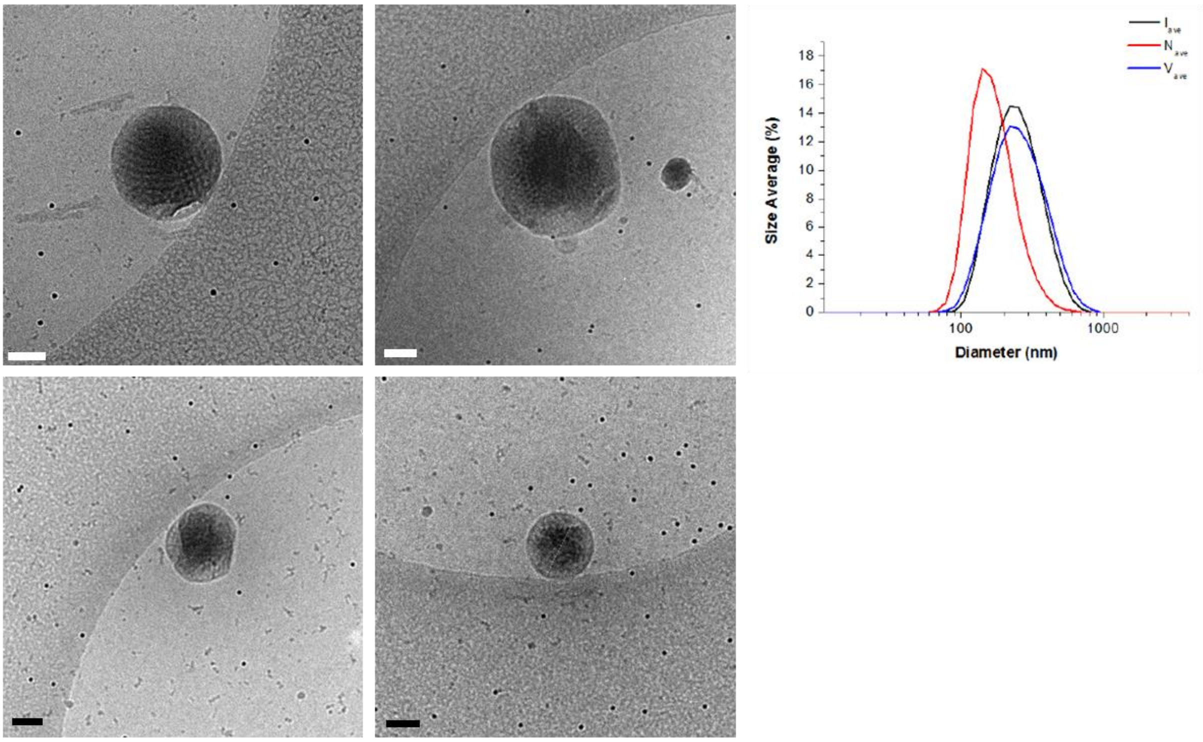

***Figure S3*:** CryoTEM images and the corresponding DLS size average curves for aggregates of PEO_{39}-*b*-$PODMA_{26}$ (f = 0.16), which forms bicontinuous nanospheres.

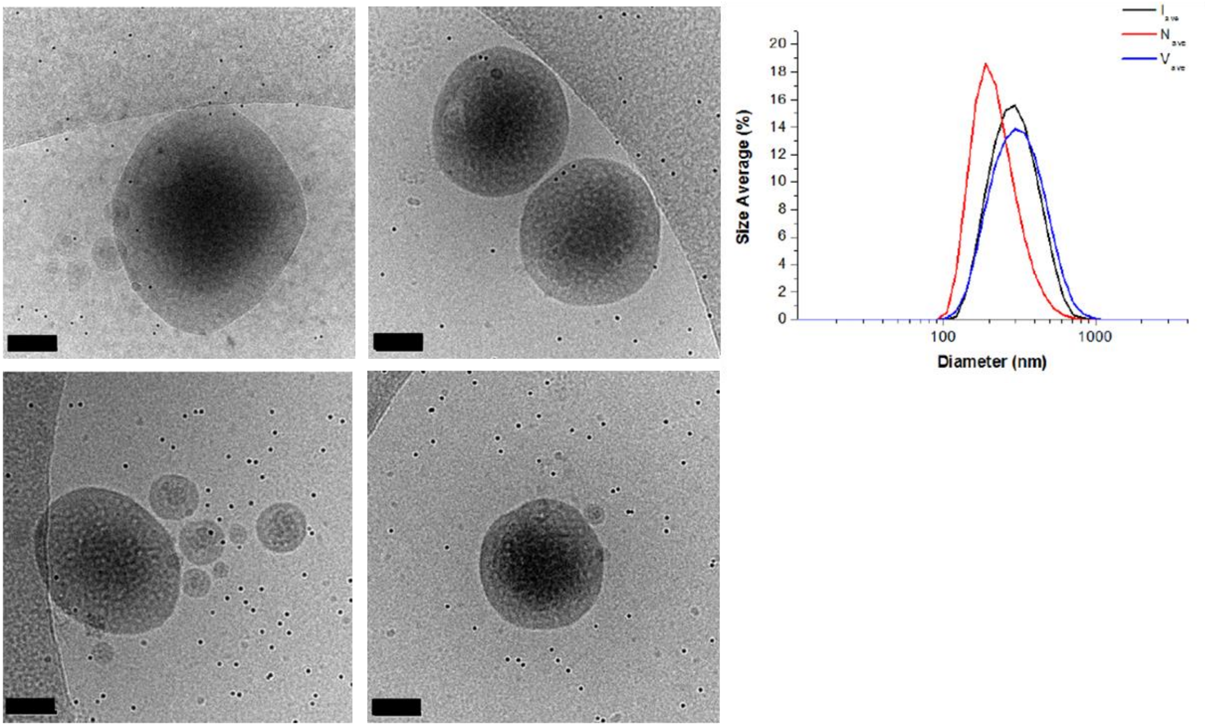

Figure S4: CryoTEM images and the corresponding DLS size average curves for aggregates of PEO_{112}-*b*-$PODMA_{15}$ (f = 0.47), which forms spherical micelles.

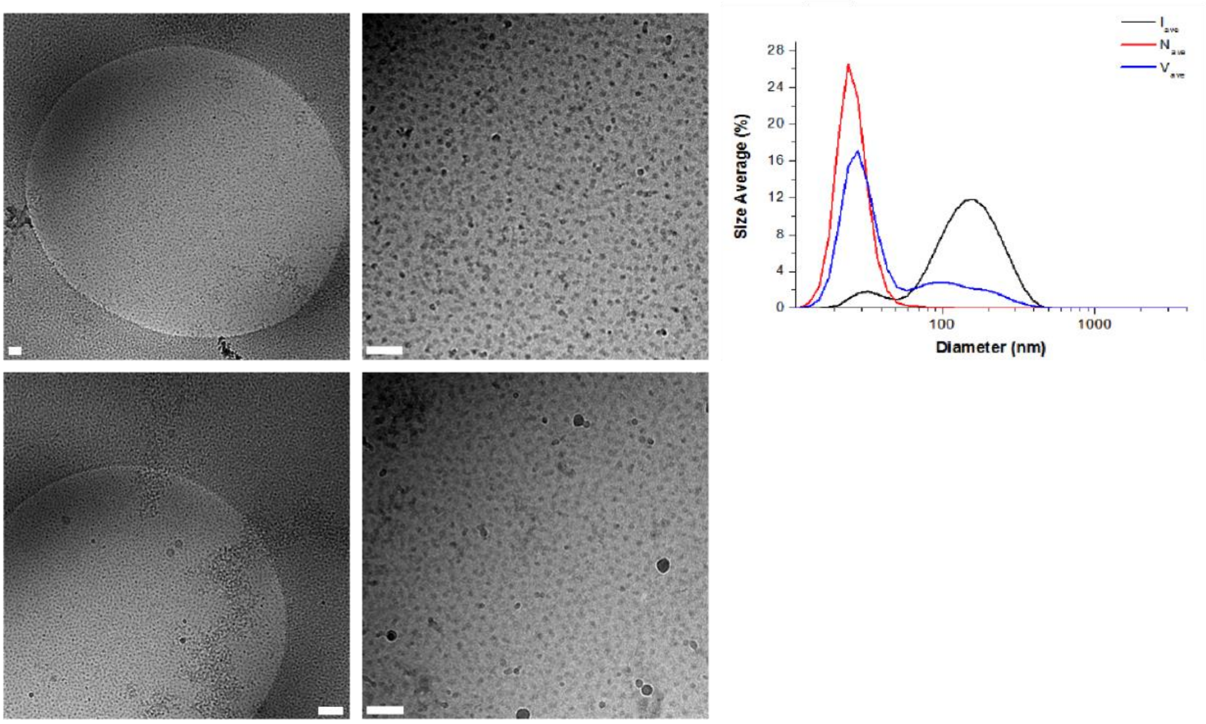

Figure S5: Negative staining TEM images and the corresponding DLS size average curves for PEO_{112}-*b*-$PODMA_{15}$ (f = 0.47) aggregates of a 0.5wt% (5 $g.L^{-1}$) dispersion, showing a mixed spherical and cylindrical micelle phase.

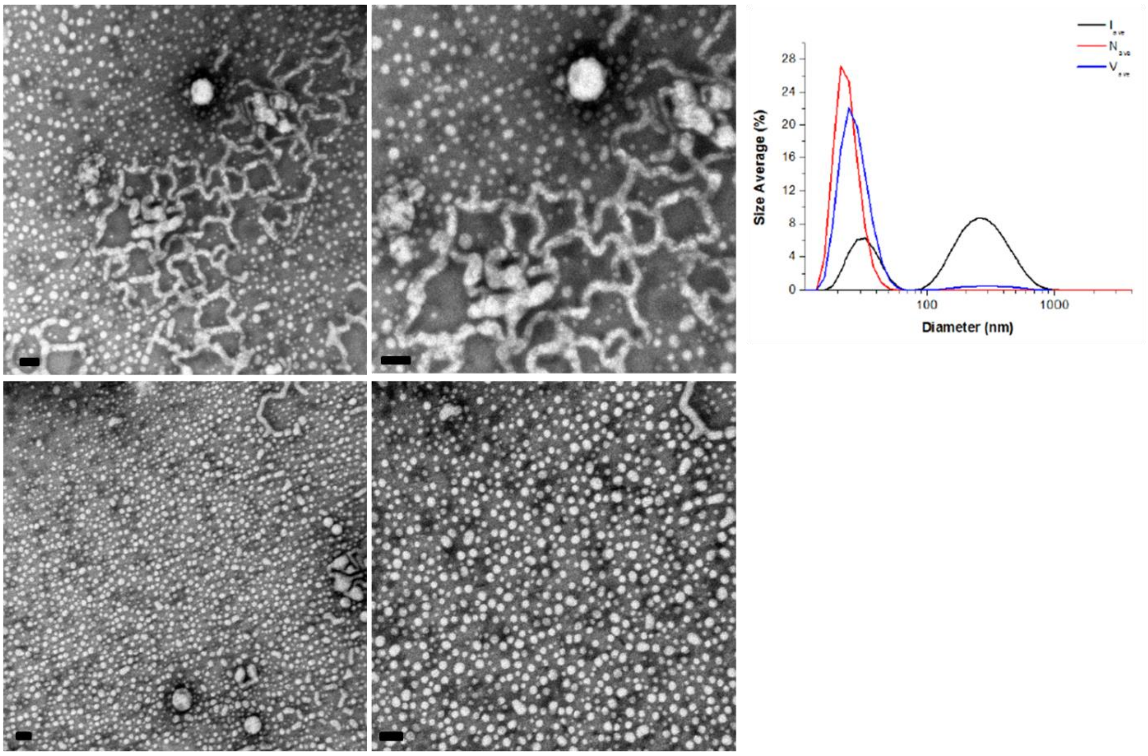

Figure S6: CryoTEM images and the corresponding DLS size average curves for aggregates of PEO$_{47}$-*b*-PODMA$_{26}$ (f = 0.22), which forms nanospheres with bicontinuous and lamellar structure.

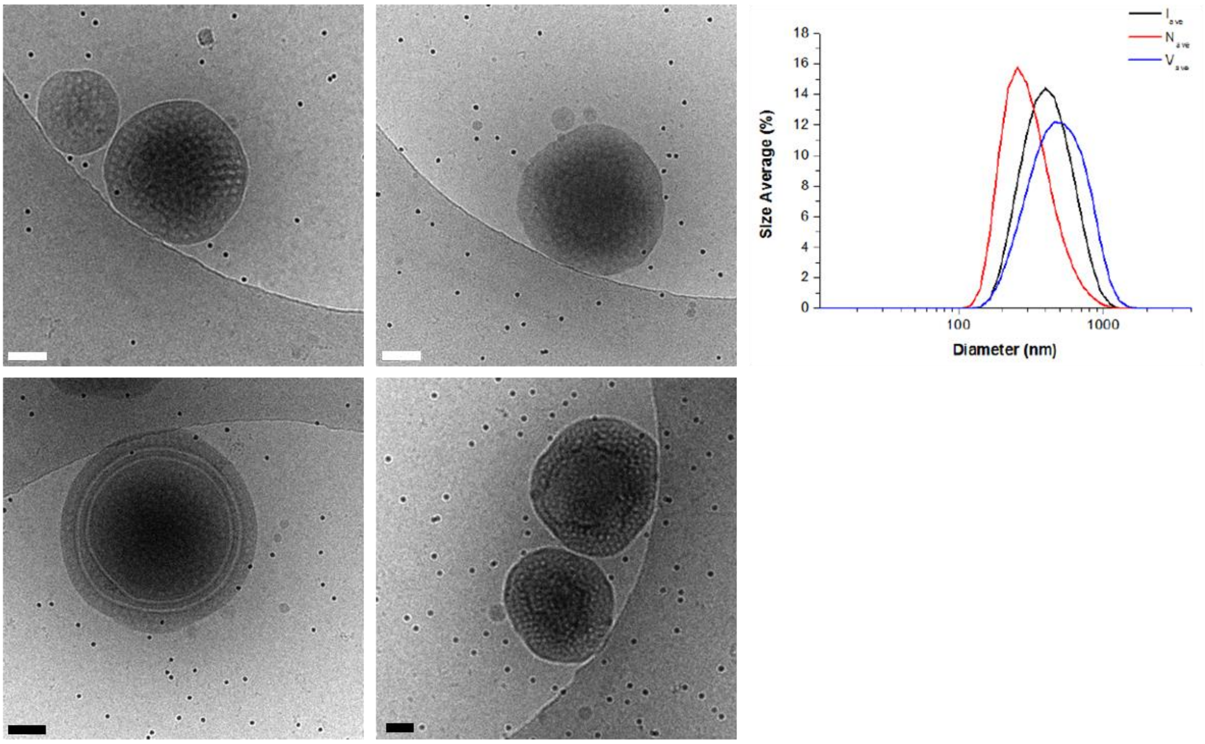

Figure S7: CryoTEM images and the corresponding DLS size average curves for aggregates of PEO_{47}-*b*-$PODMA_{20}$ ($f = 0.25$), which forms bicontinuous nanospheres.

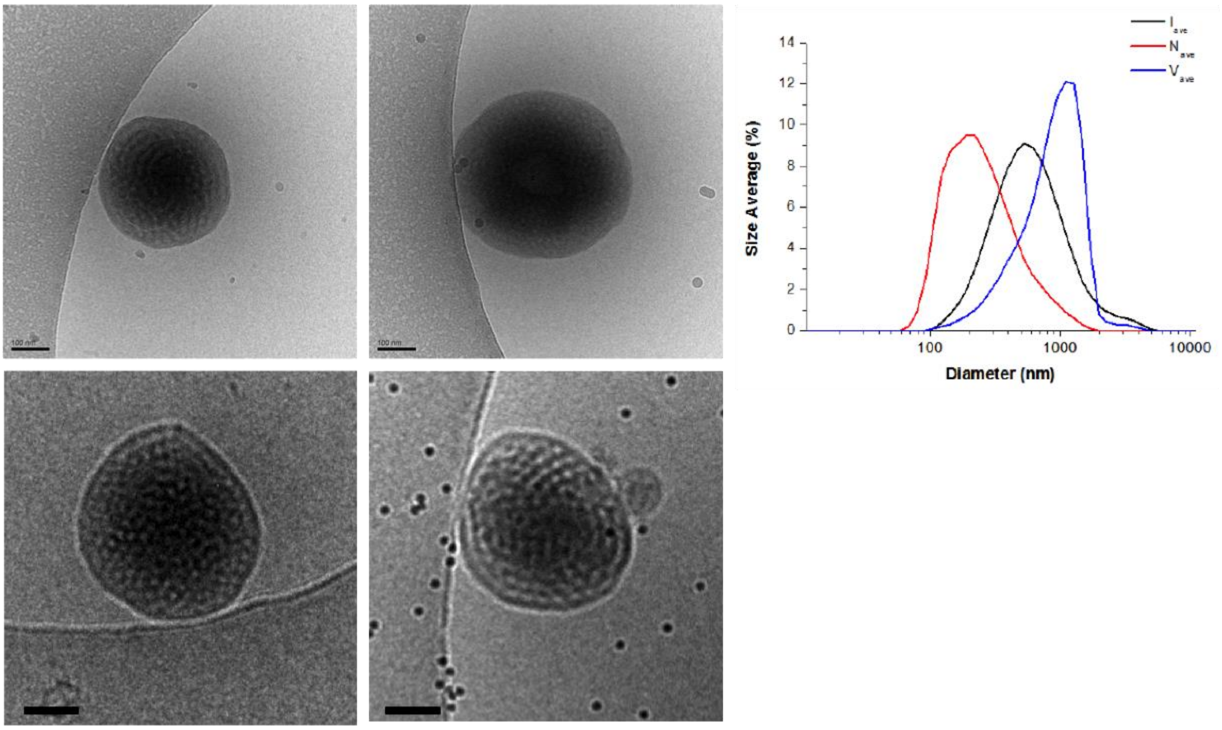

Figure S8: CryoTEM images of BPNs from PEO_{47}-*b*-$PODMA_{20}$ **A**) prepared with 78 wt% THF (as opposed to 37 wt% in Figure S7) and **B**) 20 months post preparation, with DLS curves. No changes in morphology were observed.

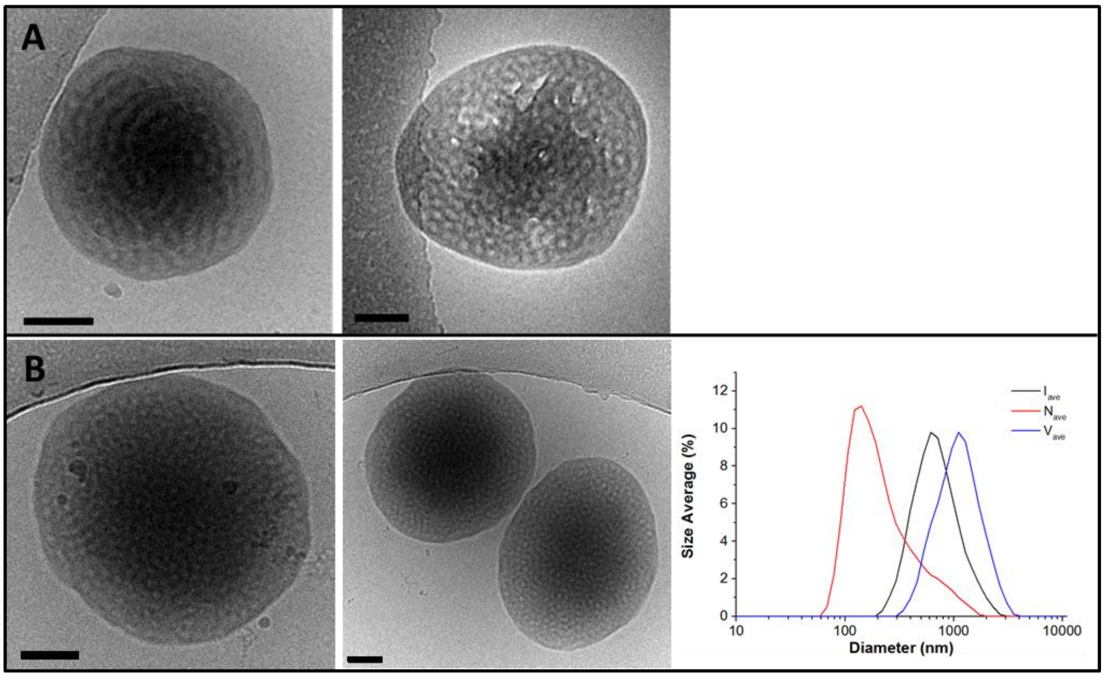

Figure S9: CryoTEM images and the corresponding DLS size average curves for aggregates of PEO_{47}-*b*-$PODMA_{13}$ (f = 0.34), which forms nanospheres with bicontinuous and lamellar structure.

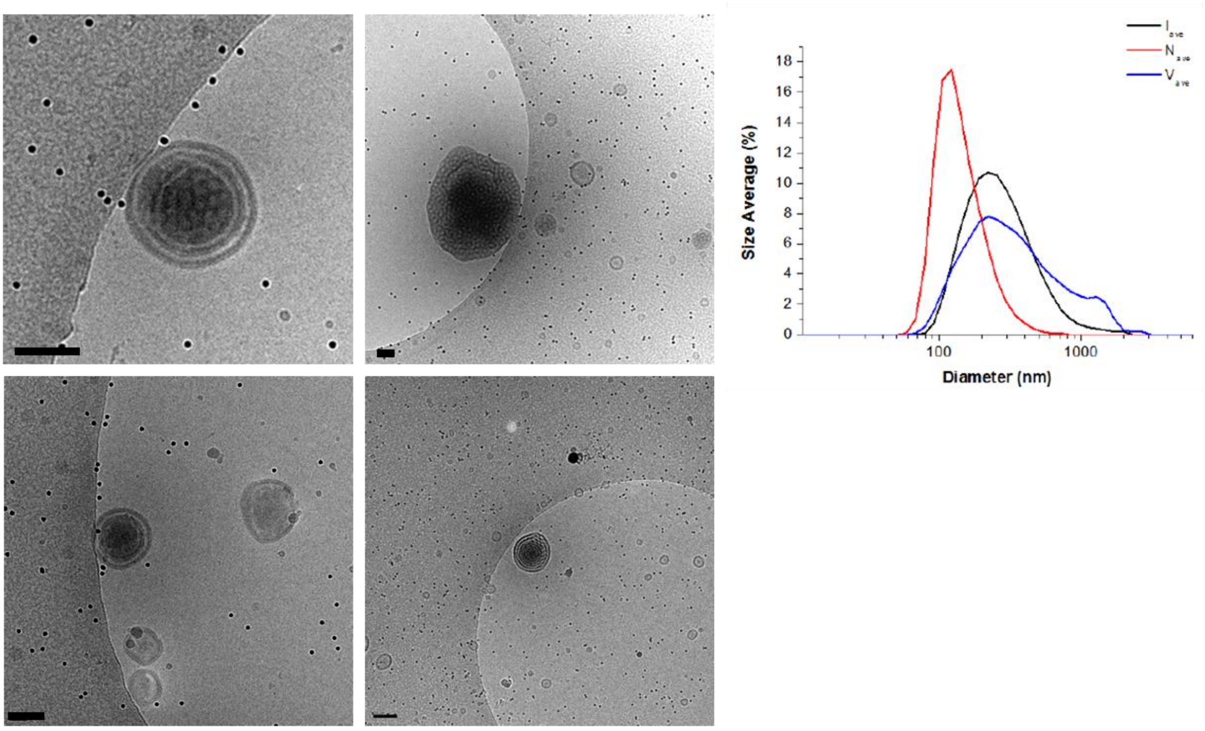

Figure S10: CryoTEM images and the corresponding DLS size average curves for aggregates of PEO_{49}-*b*-$PODMA_{21}$ (f = 0.22) at 0.1, 1 and 4 wt%, which forms bicontinuous nanospheres.

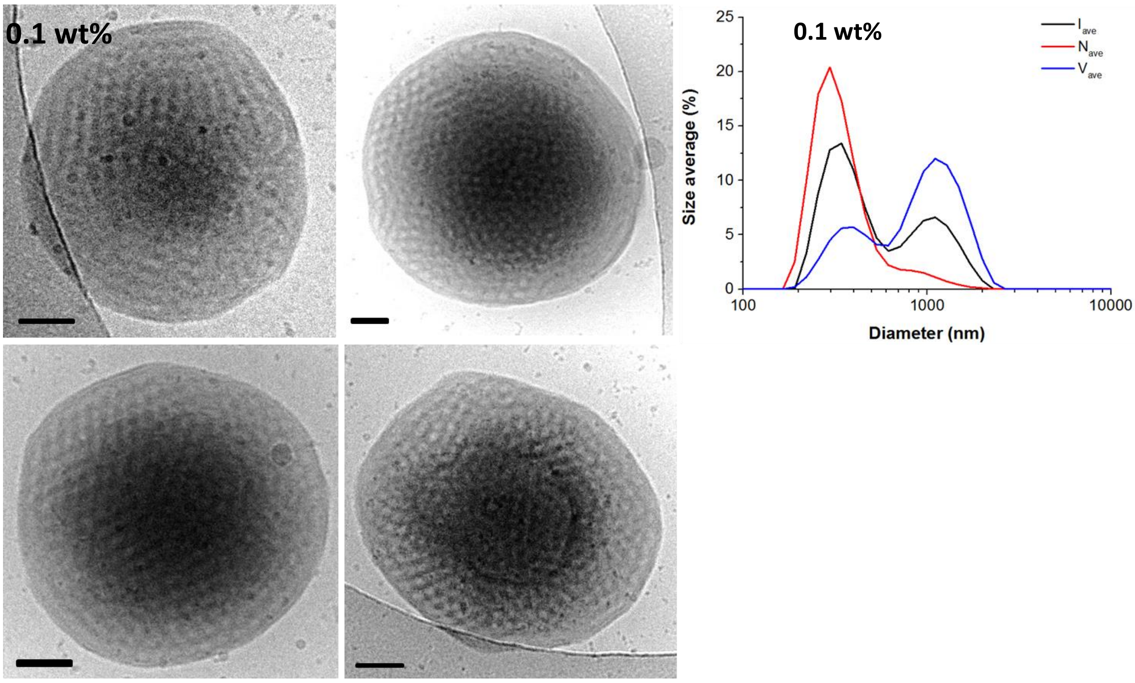

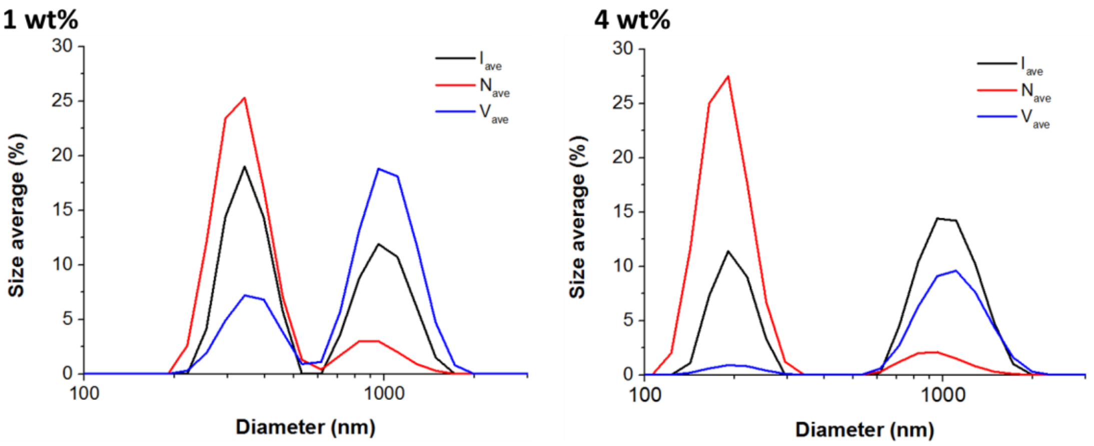

Figure S11: CryoTEM images and the corresponding DLS size average curves for aggregates of PEO_{52}-*b*-$PODMA_{26}$ (f = 0.22), which forms bicontinuous nanospheres.

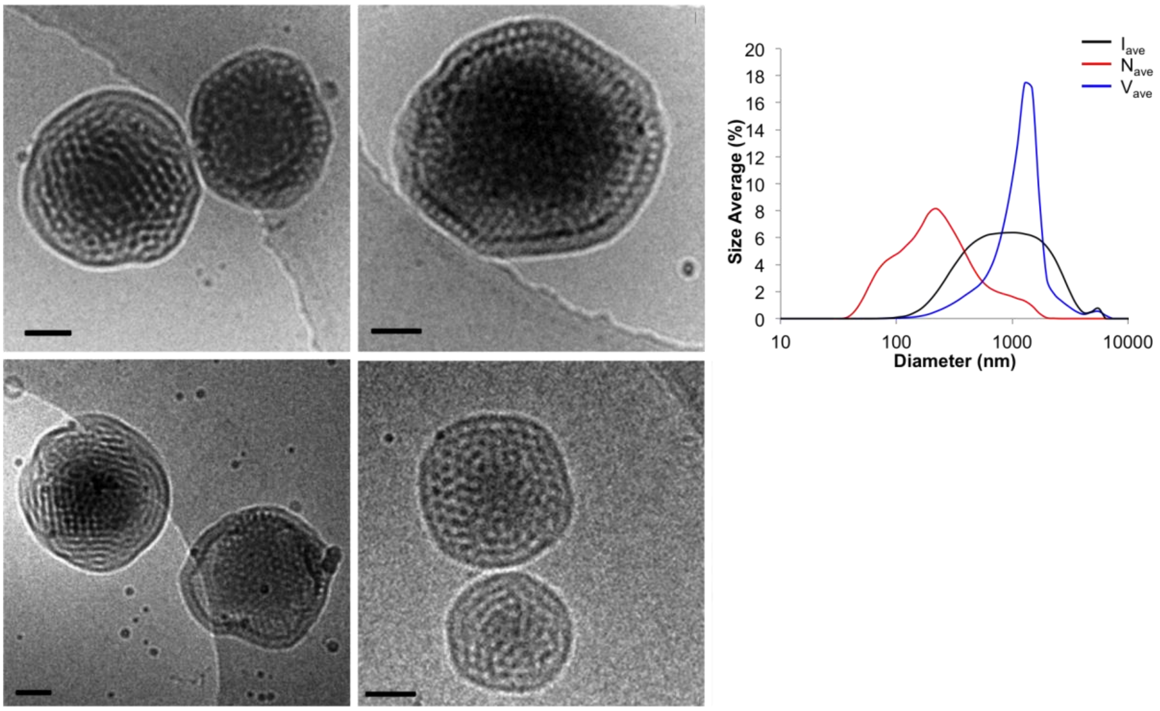

***Figure S12*:** CryoTEM images and the corresponding DLS size average curves for aggregates of PEO_{127}-*b*-$PODMA_{59}$ (f = 0.23), which forms large compound vesicles and vesicular structures.

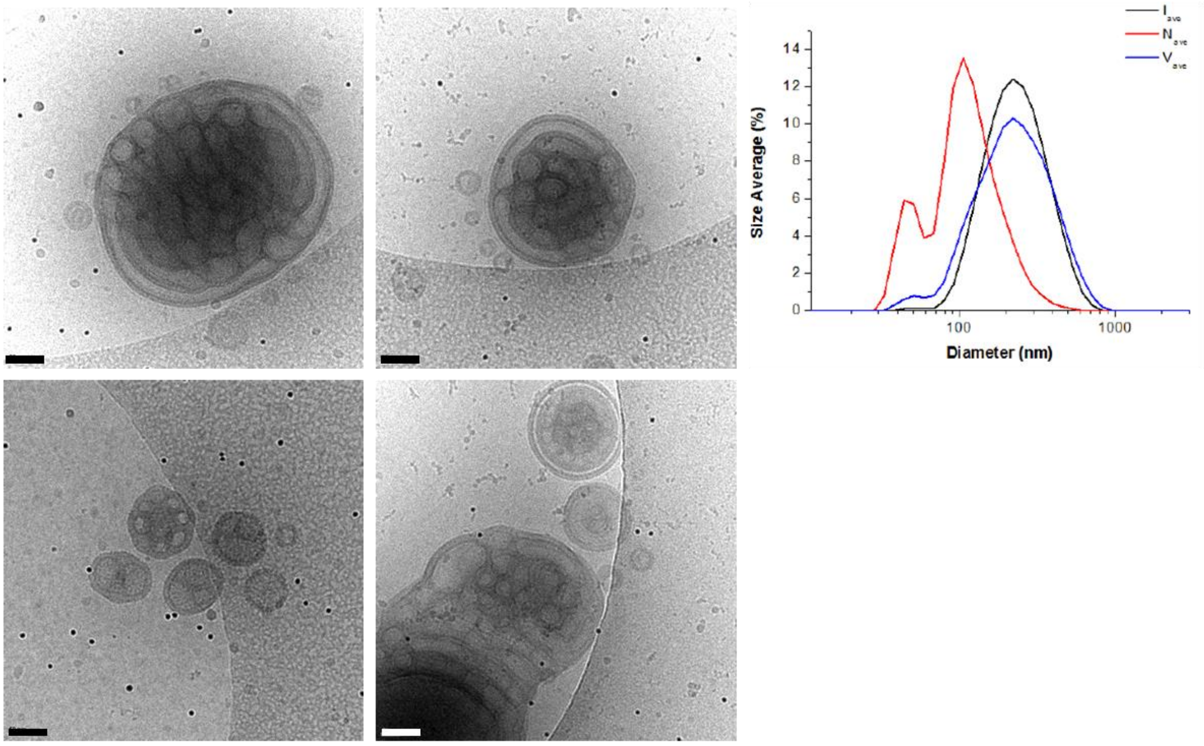

Figure S13: CryoTEM images and the corresponding DLS size average curves for aggregates of PEO_{127}-*b*-$PODMA_{38}$ (f = 0.31), which forms large compound vesicles and vesicular structures.

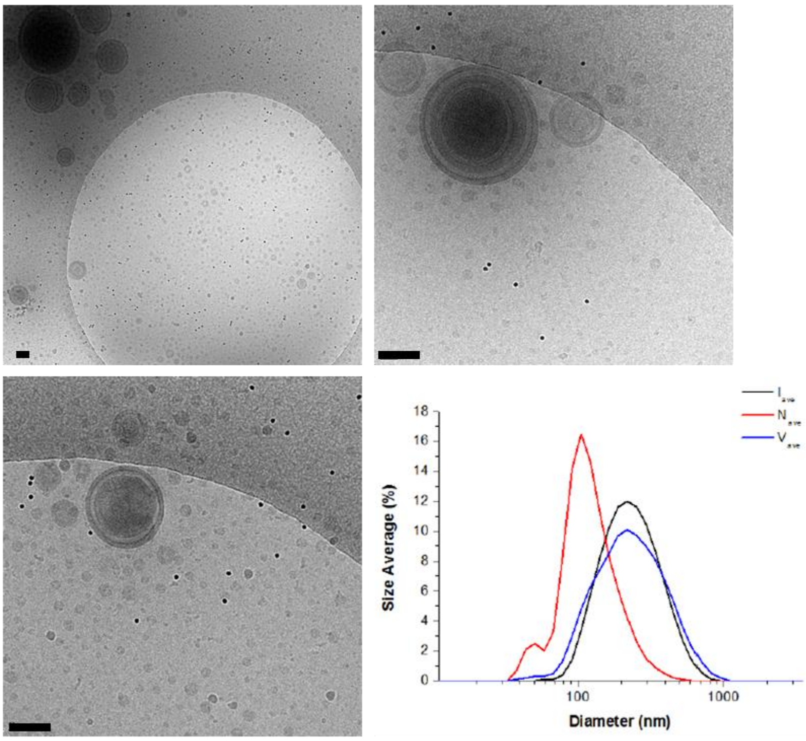

3D Cryo Electron Tomography

Figure S14: Galleries of select *z*-slices of 3D reconstructions of tilt series of aggregates from PEO-PODMA block copolymers with varied PEO contents (numbers stated in each gallery below), revealing the internal structure. All scale bars represent 100 nm.

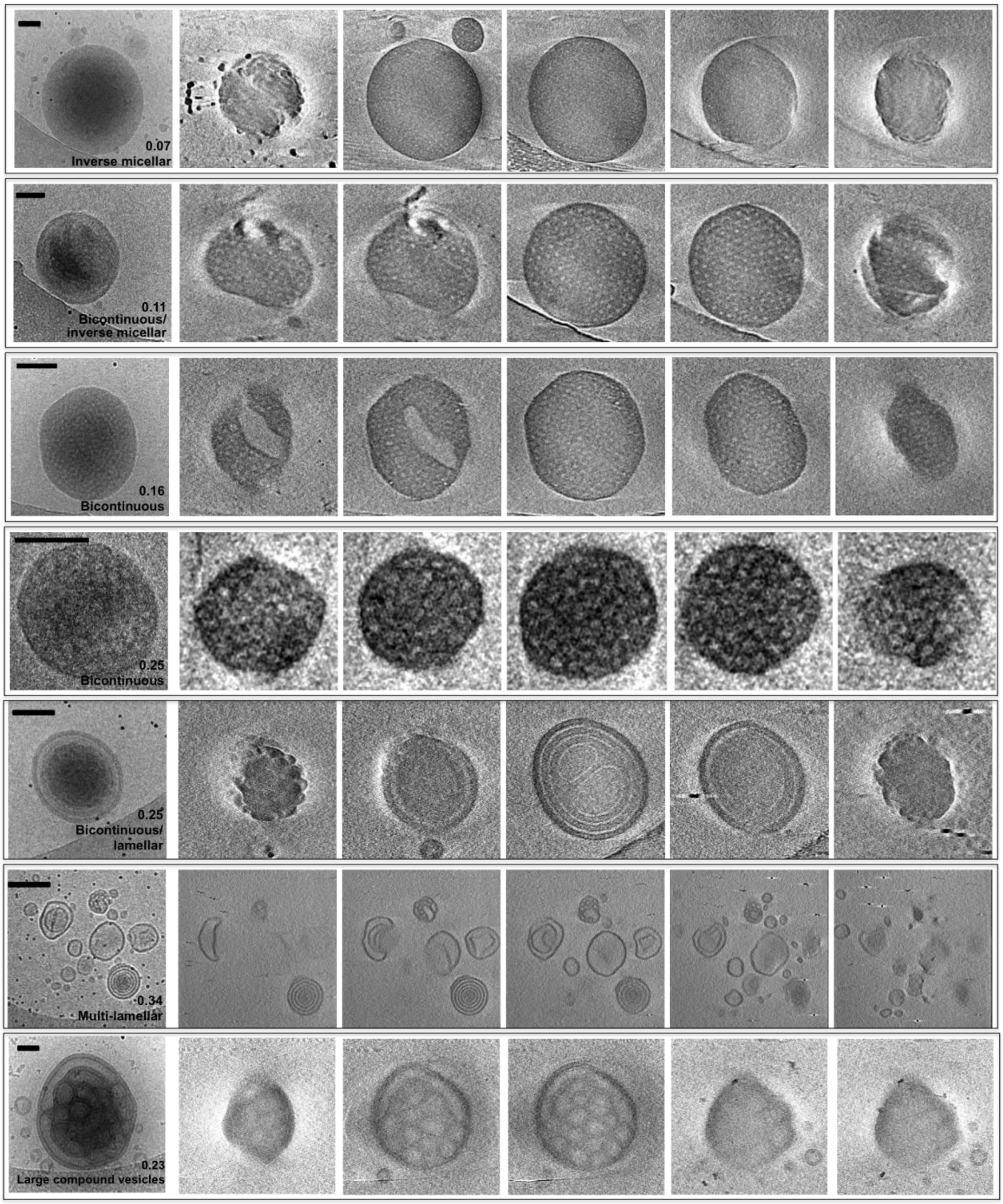

Segmentations

Figure S15: Segmentations of the reconstructed tomograms of different morphologies, with corresponding cuts through the volume (from Figure 2 in manuscript).

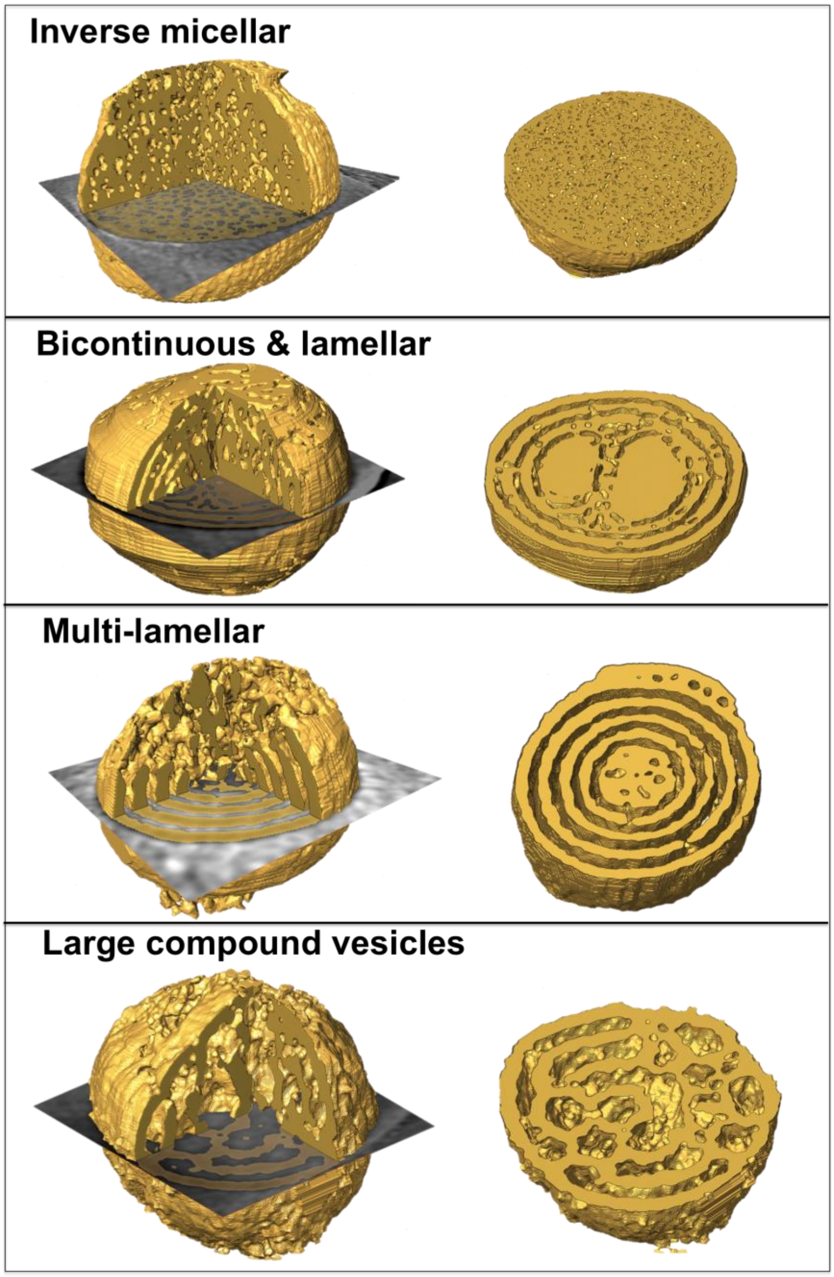

Figure S16: Segmentations of the reconstructed tomograms of bicontinuous nanospheres from block copolymers with different PEO weight fractions (*f*), with corresponding cuts through the volume (from Figure 3 in manuscript).

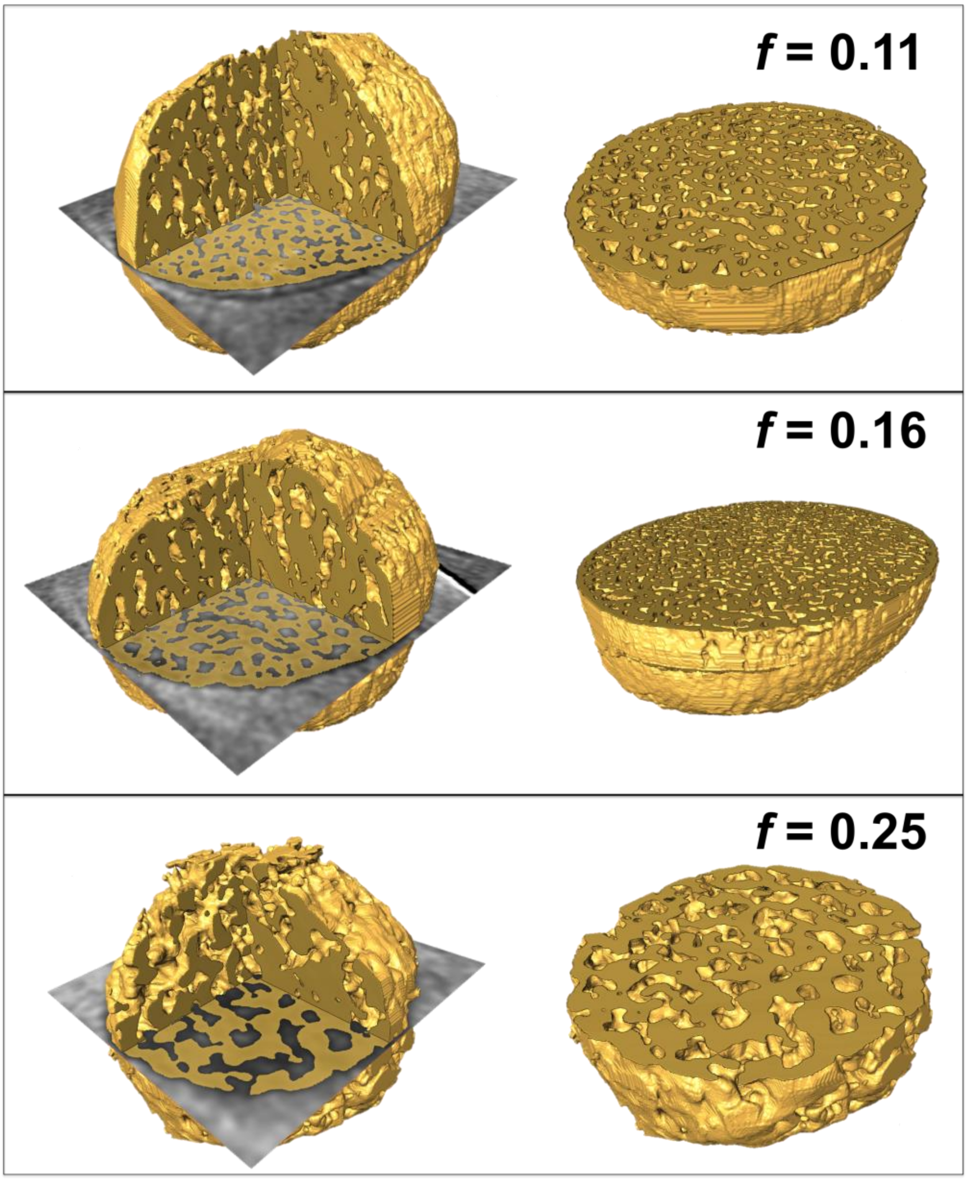

Domain size measurements

Measurements for the internal PODMA and hydrated PEO regions were obtained by analysing the 3D reconstructions of the aggregates using in-house MATLAB scripts. The PODMA wall thickness was defined as the shortest distance between two neighbouring bright aqueous channels when browsing up and down through the *xy*-slices of the tomogram. Measurements were obtained from an average of at least 20 points in the PODMA wall. Equivalently, pore size measurements were performed by finding the widest diameter of bright aqueous regions when browsing up and down through the *xy*-slices of the tomogram. An illustration of the process is provided below in Figure S15.

Figure S17: *xy*-slices of 3D reconstructions of tilt series of aggregates from PEO_{39}-*b*-$PODMA_{47}$ (11wt% PEO) with markings for the measurements of **A)** the PODMA wall thicknesses and **B)** and **C)** the PEO pores. For pore sizes, the long and short axis (red and yellow lines respectively) were measured and the average taken.

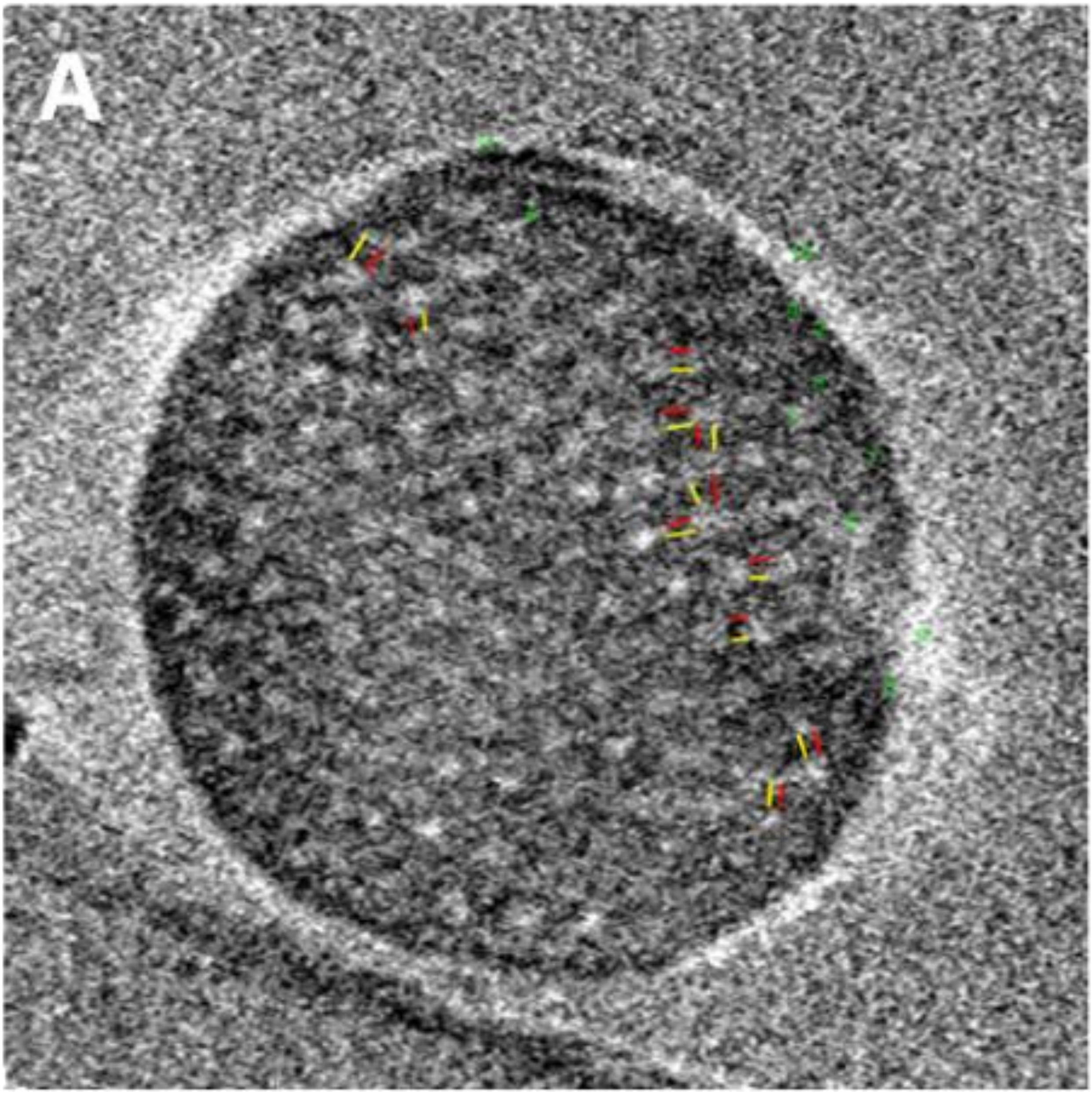

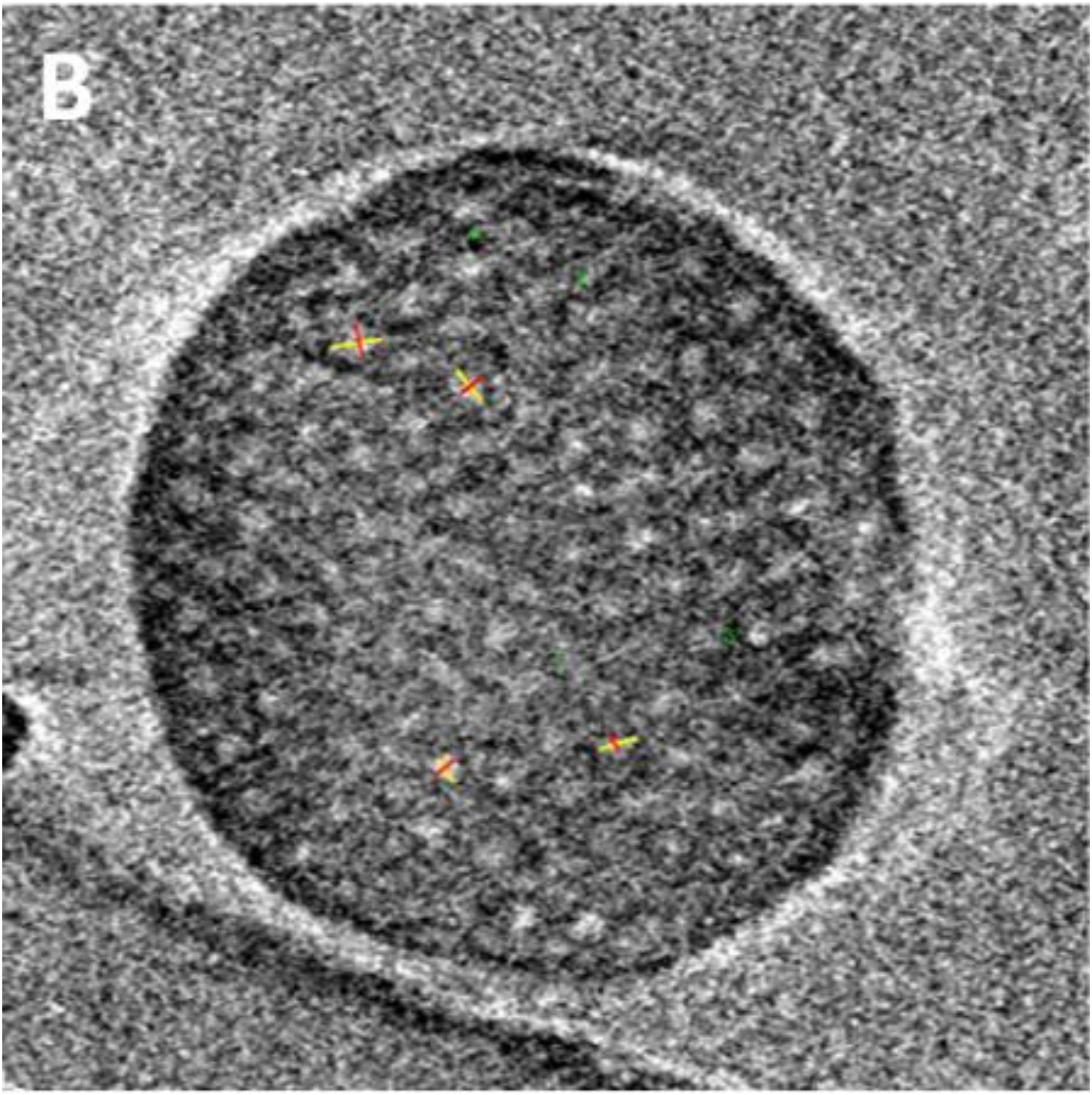
B

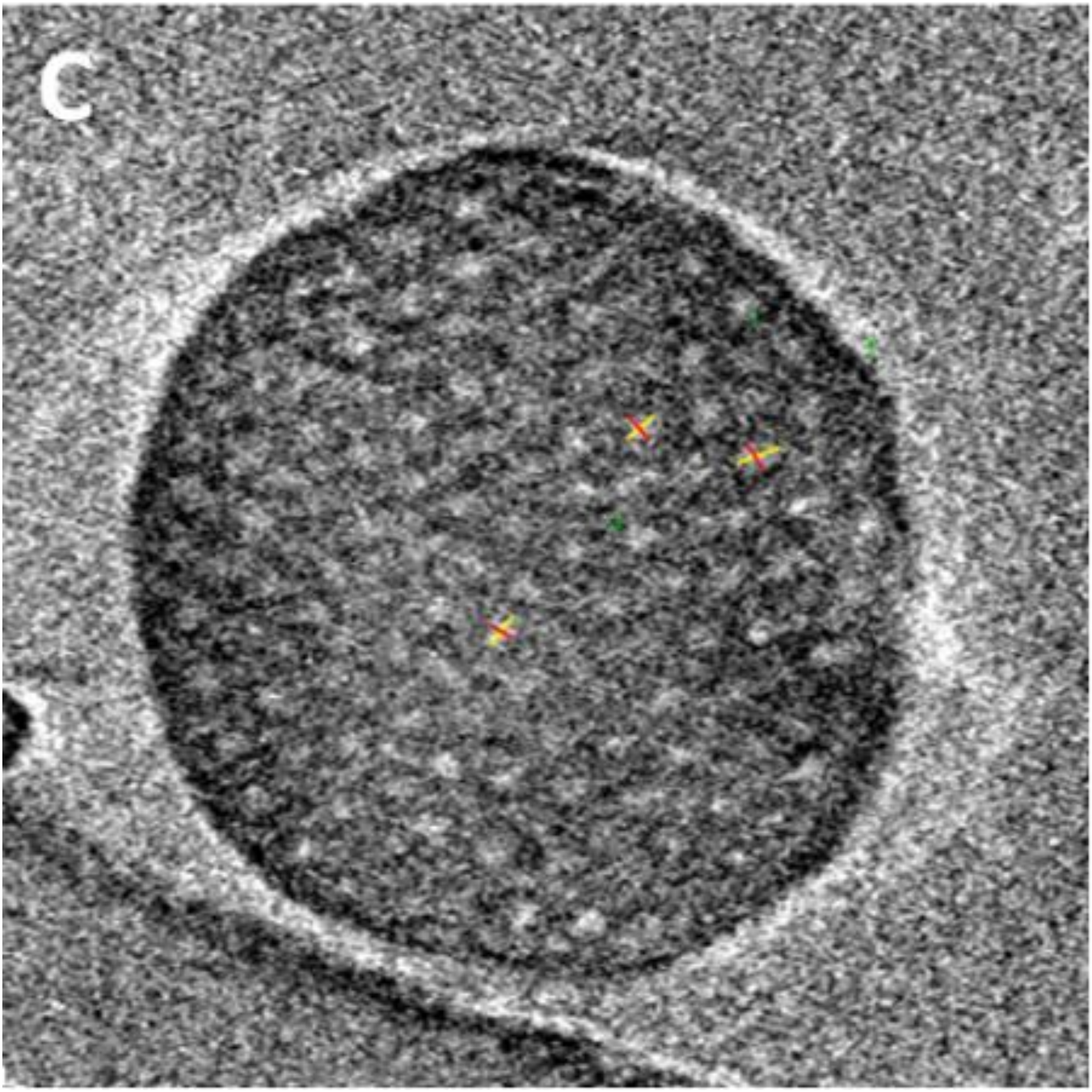
c

Figure S18: Thickness of the PODMA wall domain as a function of the degree of polymerization in the PODMA block (DP_{PODMA}).

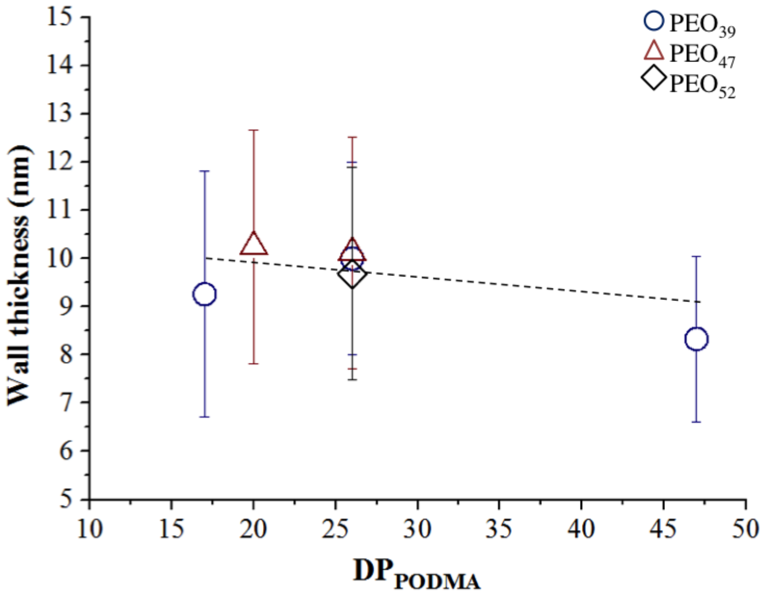

Figure S19: Representative negative staining TEM images of the nanospheres prepared from PEO_{47}-*b*-$PODMA_{20}$ at different starting volumes of THF.

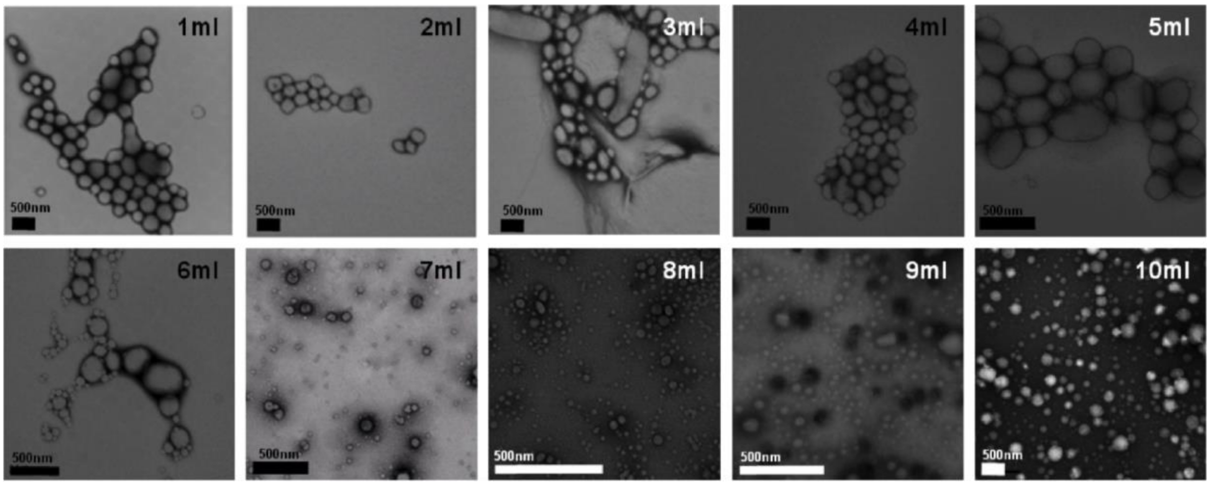

References

[1] a) G. Street, D. Illsley, S. Holder, *J. Polym. Sci. Pol. Chem.* **2005**, *43*, 1129-1143; b) D. M. Haddleton, C. B. Jasieczek, M. J. Hannon, A. J. Shooter, *Macromolecules* **1997**, *30*, 2190-2193.

[2] J. R. Kremer, D. N. Mastronarde, J. R. McIntosh, *J. Struct. Biol.* **1996**, *116*, 71-76.

[3] A. S. Frangakis, R. Hegerl, *J. Struct. Biol.* **2001**, *135*, 239-250.

[4] K. Jankova, X. Chen, J. Kops, W. Batsberg, *Macromolecules* **1998**, *31*, 538-541.
